# On the impact of re-mating and residual fertility on the Sterile Insect Technique efficacy: Case study with the medfly, *Ceratitis capitata*

**DOI:** 10.1371/journal.pcbi.1012052

**Published:** 2024-05-06

**Authors:** Yves Dumont, Clélia F. Oliva

**Affiliations:** 1 UMR AMAP, CIRAD, Saint-Pierre, Réunion island, France; 2 UMR AMAP, Univ Montpellier, CIRAD, CNRS, INRAE, IRD, Montpellier, France; 3 Department of Mathematics and Applied Mathematics, University of Pretoria, Pretoria, South Africa; 4 CTIFL, Bellegarde, France; The Hebrew University of Jerusalem, ISRAEL

## Abstract

The sterile insect technique (SIT) can be an efficient solution for reducing or eliminating certain insect pest populations. It is widely used in agriculture against fruit flies, including the Mediterranean fruit fly (medfly), *Ceratitis capitata*. The re-mating tendency of medfly females and the fact that the released sterile males may have some residual fertility could be a challenge for the successful implementation of the SIT. Obtaining the right balance between sterility level and sterile male quality (competitiveness, longevity, etc) is the key to a cost-efficient program. Since field experimental approaches can be impacted by many environmental variables, it is difficult to get a clear understanding on how specific parameters, alone or in combination, may affect the SIT efficiency. The use of models not only helps to gather knowledge, but it allows the simulation of a wide range of scenarios and can be easily adapted to local populations and sterile male production.

In this study, we consider single- and double-mated females. We first show that SIT can be successful only if the residual fertility is less than a threshold value that depends on the basic offspring number of the targeted pest population, the re-mating rates, and the parameters of double-mated females. Then, we show how the sterile male release rate is affected by the parameters of double-mated females and the male residual fertility. Different scenarios are explored with continuous and periodic sterile male releases, with and without ginger aromatherapy, which is known to enhance sterile male competitiveness, and also taking into account some biological parameters related to females that have been mated twice, either first by a wild (sterile) male and then a sterile (wild) male, or by two wild males only. Parameter values were chosen for peach as host fruit to reflect what could be expected in the Corsican context, where SIT against the medfly is under consideration.

Our results suggest that ginger aromatherapy can be a decisive factor determining the success of SIT against medfly. We also emphasize the importance of estimating the duration of the refractory period between matings depending on whether a wild female has mated with a wild or sterile male. Further, we show the importance of parameters, like the (hatched) eggs deposit rate and the death-rate related to all fertile double-mated females. In general, re-mating is considered to be detrimental to SIT programs. However, our results show that, depending on the parameter values of double-mated females, re-mating may also be beneficial for SIT.

Our model can be easily adapted to different contexts and species, for a broader understanding of release strategies and management options.

## Author summary

The Sterile Insect Technique (SIT) is an old, but still challenging, method to control insect pest and disease-vector by releasing sterile males within a wild population. Mating between sterile males and wild females usually results in no progeny, and a progressive reduction of the wild population. Several biological parameters can impact the success of SIT. We study the impact of male residual fertility and female re-mating on the efficacy of SIT to control a (fruit) fly population. We prove the existence of a relationship between the residual fertility parameter and the parameters related to single- and double-mated fertile females to ensure the existence of a strong Allee effect related to SIT. Using numerical simulations, we study several cases. In particular, we show that re-mating is not necessarily detrimental to the SIT success. Our results emphasize the importance of some parameters that are not systematically well studied in SIT programs against fruit fly.

## Introduction

The Sterile Insect Technique (SIT) is an established autocidal control method genuinely used against several agricultural pests in many countries worldwide. Some of the major operational programs include the Mediterranean fruit fly (medfly) *Ceratis capitata*. SIT relies on a continuous mass production of the targeted insect, the sterilization of males (or both males and females, according to the species) as pupae or adults, using ionizing radiation, and their repeated and massive releases in the field resulting in a progressive decay of the targeted pest population [1–3].

Ionizing radiation is now the most common method used to sterilize males as part of operational programs. In order to release only males, and reduce the mass-rearing costs, it is important to have an efficient method to separate males and females at an early stage. To this end, a genetic sexing strain (GSS) was developed in the nineties for medfly [4], based on temperature sensitivity. This development has permitted several countries to benefit from an economically viable tool to control medflies. Unfortunately, GSS strains are not available for most insects for which SIT is being used, as its development can be technically challenging. However, improvements are being studied, see for instance [5]. SIT is never used alone, but as a primary control program in several operational integrated pest management (IPM) programmes that use additional suppression methods, like insecticides and mass-trapping, or other cultural practices, etc. See for instance [6].

A genetically engineered sterile strain of medfly (*C. capitata*) has been developed and tested for population reduction on a small scale [7], as an alternative to SIT. Other suppression approaches include the incompatible insect technique (IIT), which uses the bacterial endosymbiont Wolbachia [8], have been considered against Medflies [9]. In the present study, we consider only the approach based on sterilization with irradiation.

Conceptually, the Sterile Insect Technique is simple, but its efficient deployment requires understanding and tailoring several technical, logistical, ecological, and biological parameters, as well as socioeconomic elements [2]. Although the use of SIT against medflies is widespread (it is used in South and Central America, USA, Australia, South Africa, etc.), in Europe it is only operational in Spain and Croatia. France is developing an SIT research pilot project to determine the optimal conditions for the deployment of the SIT on Corsica Island to protect stone fruits and clementine production (CeraTIS Corse project).

The Corsican agricultural context is a mosaic of small plots with different cultivars that span the harvest over several months. The climate is particularly suitable for medflies, which are found in traps from May to December, with low adult levels potentially hibernating and reproducing in pomelos as early as February. Thus, the Mediterranean fruit fly is the dominant fruit fly pest on citrus and deciduous fruit in Corsica, where current treatments still rely mostly on the use of pesticides. The growing demand for more environmentally friendly approaches, together with the potential future unavailability of chemical substances, triggered a pilot research project to integrate SIT in the control strategies. Most Corsican orchards have challenging initial ecological conditions (high density of flies, crop areas surrounded by rural settings, and wild host plants for medflies). Designing an efficient and cost-effective suppression program requires a good understanding of the biological and technical parameters that impact its success. As part of this effort, modeling the effect of sterile male residual fertility, female re-mating and release frequency on release ratios is essential.

SIT impacts the reproduction of the targeted insect. Hence, its success depends on the ability of the released sterile males to find a lek, to perform courtship, to be selected by wild females, to successfully copulate and inseminate females with their sterile sperm, while eliciting effective female refractoriness to further re-mating by wild males. Medflies are considered to have a complex courtship behavior that could make SIT less efficient [10]. Nevertheless, the SIT is a well established method of suppressing the population of medflies and has been proven highly efficient and economically viable in many applications [11]. Modeling, model analysis, and simulations can be helpful in highlighting the positive or negative impact of specific parameters and assist in formulating an optimal release strategy in the field.

SIT has been modeled since the early work by Knipling [1]. Various models have been developed of varied complexity depending on the number of stages in the life of the targeted pest/vector: for instance probabilistic models [12], computer models [13, 14], discrete models [15–17], semi-discrete models [18–20] or continuous models [21–24], using sometimes tools from control theory [25–27] to optimize the release protocols. Many SIT models consider sterile males to be fully sterilized, although this is rarely achieved. This is because the sterility (expressed as unhatched eggs) of irradiated male pupae gradually increases as the radiation dose increases. Thus, reaching full sterility requires a very high dose of radiation [28, 29], which often impairs the quality (competitivity, flight ability, etc) of males to a level that is not acceptable. When all sterile males are not 100% sterile, it means that they are able to produce, on average, a certain quantitity of viable (hatched) eggs. The corresponding proportion is called the residual fertility. As a result, at the population level, this is equivalent to consider that a small percentage of the sterile males fertilize wild females and, thus, produce viable (hatched) eggs. In other words, releasing a proportion of almost-sterile males is equivalent, in these conditions, to releasing a certain reduced proportion of fertile males.

Residual fertility is easy to assess and is part of the quality control of every SIT program (interested readers are referred to the manual published by the International Atomic Energy Agency (IAEA) [30] for medfly SIT procedures). For *C. capitata*, a dose of 140Gy is required to achieve full sterility, as implemented in the medfly management program in Argentina [31]. However, this dosage can also negatively affect male performance and therefore be detrimental for SIT operations [32, 33]. On the other hand, in an ongoing operational SIT program against medflies in the region of Valencia, Spain, the induced average sterility reached 98.87 ± 0.55% with an irradiation dose of 100 Gy, leading to a residual fertility of 1.13 ± 0.55% [6]. This program is still running with good results.

Therefore, it is important to assess the threshold above which residual fertility can have a negative impact on SIT program performance. In a recent study, on a different fruit fly species [20] and considering single mating only, we investigated this question using a minimalistic model. The result was straightforward: the percentage of residual fertility, *ε*, must be lower than $\frac{1}{N}$, where N is the basic offspring number, also called the basic reproduction number, which is related to the reproductive potential of the pest population. Clearly, for a wild population with a large N, the constraint on residual fertility can be strong, thereby significantly reducing its acceptable level for successful SIT. A similar result was obtained by Van den Driessche [15] using discrete models. In the present study, we explore a more complex model, with single and double mating, to verify these results, with a particular focus on medfly. Furthermore, we consider the important parameter of re-mating and whether there is a different response when wild females mate with sterile males as opposed to wild males.

Female medflies are facultatively polyandrous [34, 35]. After mating, medfly females typically exhibit an average refractory period of two and a half days [36]. Their propensity to remate can be triggered by the courtship behavior of the male, sexual performance, and amount of ejaculate transferred [37–39]. The likelihood of re-mating might (as evaluated under laboratory conditions) increase in situations of high availability of oviposition substrate or highly male-biased sex ratios, which is the case of male-only release SIT programs [36, 40].

Medfly females possess 2 spermathecae, spherical organs that store sperm after insemination, and a fertilization chamber that serves as a functional third spermatheca [41]. Having more than one spermatheca could be seen as advantageous in case of multiple insemination. In most insects, the sperm from different males is mixed within the spermathecae, therefore not physically allowing for preferential selection during egg fertilization. However, the fertilization chamber in medflies allows a second male to remove sperm from the previous one [41]. Sperm precedence of the second male mating a female tends to succeed in fertilizing eggs (second male contribution greater than 0.5) [34, 39, 42–44]. In [45], the authors studied double-mated females and show how the sperm of the first and the second mating are stored, meaning that both are able to be used to fertilize the eggs. In [44, 46], the authors show that re-mating increases the fitness of females, while Katiyar et al. [43] showed that the copulation order between fertile and sterile males impacts the fitness of females, with a precedence of the second sperm. Lee et al. [42] reported that male genotype, copulation order and genotypic differences may affect the variation in sperm precedence. From the SIT point of view, this is not necessarily a good news. In the rest of the paper, for sake of simplicity, we will consider single- and double-mating, but our reasoning could be extended to more than two matings.

In the present study, we investigate how residual fertility and re-mating can impact the critical release ratio under different situations. Most medfly SIT programs involve biweekly releases, some daily releases. Although release frequency has a direct impact on the program efficiency, it is also useful to understand how this impact is affected by biological parameters. More precisely, as most programs implement the addition of ginger root oil (GRO) aromatherapy to enhance sterile male attractiveness [47–49], we include a comparison of competitiveness parameters for treated and untreated males. In the rest of the paper, we consider mostly data related to the V8 (Vienna-8) strain obtained from laboratory colonies maintained by the Seibersdorf laboratory of IAEA (International Atomic Energy Agency, Vienna, Austria). The V8-strain is a GSS-strain: it allows to separate easily males and females. In [50], the authors showed that 99% of V8 strain copulations resulted in sperm transfer, and that the V8-strain has a good re-mating potential.

The paper is organized as follows: in Section 1, we build the continuous SIT model with re-mating and residual fertility. We also derive some theoretical results before extending the model to periodic impulsive releases. In Section 2, we provide numerical simulations related to continuous and periodic releases, highlighting different re-mating cases. We discuss the results in section 3. Finally, in Section 4, we derive some conclusions and perspectives.

## 1 Material and methods

**Fig 1:**
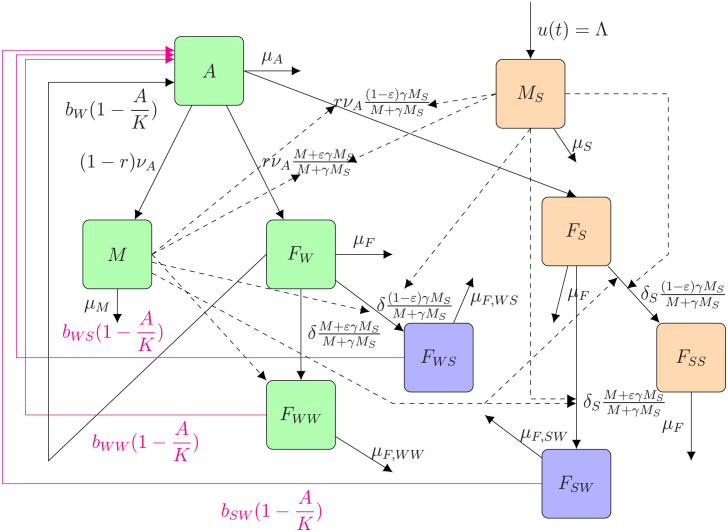
Flow diagram of model (1): the green squares represent the (fully) wild population (with females mated once or twice with wild males only); the blue squares represent the double-mated females (with fertile (sterile) and then sterile (fertile) males); the orange squares represent the sterile population (sterile males released and females mated once or twice with sterile males only).

Assuming a large population of medflies and the fact that all interactions between individuals and all biological processes occur simultaneously, we consider a continuous modeling approach, using a system of ordinary differential equations to model the biological system. In the forthcoming model, we consider several compartments (see the compartmental diagram in Fig 1, page 7): flies in immature non-flying stages, *A*, encompassing larvae and pupae; wild males, *M*; once-mated females with fertilized eggs, *F*_*W*_; once-mated females with non-fertilized eggs, *F*_*S*_; females mated twice, first by a fertile male and then by a sterile one (*F*_*WS*_); first by a sterile male and then by a fertile one (*F*_*SW*_); twice by a sterile male (*F*_*SS*_) or twice by a fertile male (*F*_*WW*_); “almost” sterile males, *M*_*S*_. We also assume that there is no migration of fruit flies from outside the treated zone, that is the biological system is isolated. The females *F*_*S*_ and *F*_*SS*_ are assumed to be fully sterile, ie. to have a fully sterile progeny. We consider that no sterilized females are being released, as the use of the GSS strain allows removing them from early stages.

We consider different birth-rates and death-rates for each (fertile) female compartments, that is *b*_*W*_, *b*_*WW*_, *b*_*WS*_, *b*_*SW*_. In particular, following [46], we have *b*_*WW*_ ≥ *b*_*W*_ and *μ*_*F*,*WW*_ < *μ*_*F*_: re-mated females have greater longevity and higher productivity than one-time maters. Concerning the birth and death rates of the females in the compartments *F*_*SW*_ and *F*_*WS*_, we have some scarce knowledge [42, 43], but nothing on μ_*_. In the numerical part, we will make several simulations to show the importance of knowing these parameters.

We model the residual fertility like in [20, 51, 52]: we assume that in the sterile male population there is a small proportion, *ε*, of fertile sperm, so that at the population-level, we assume that a proportion of *ε* sterile males is able to fertilize females. All variables and parameters are described in Table 1, page 6. As seen in Fig 1, page 7, the birth rates are impacted by $1-\frac{A}{K}$, where *K* is the carrying capacity (i.e. maximum number of larvae/pupae for all fruits) of the host(s). Since the average mating rate at first mating was shown to be similar for wild and V8 sterile males (0.67 and 0.64, respectively, [50]), we consider equal chance for wild (sterile) males to mate. Without residual fertility, the impact of SIT is modeled by the term $\frac{M}{M+\gamma M_{S}}$ ($\frac{\gamma M_{S}}{M+\gamma M_{S}}$, respectively) which represents the probability for a sexually mature female to mate with a wild (sterile, respectively) male and enter one of the mated female compartments, *F*_*W*_ (*F*_*SW*_, *F*_*W*_) or *F*_*WW*_ (*F*_*S*_). Male residual fertility is modeled by considering that a proportion, *εM*_*S*_, of sterile males is fertile, so that emerging immature females will become fertile with a probability of $\frac{M+\gamma \varepsilon M_{S}}{M+\gamma M_{S}}$ or sterile with a probability of $\frac{\left(1-\varepsilon \right)\gamma M_{S}}{M+\gamma M_{S}}$.

**Table 1:** Description of parameters and state variables of model (1).

Symbol	Description	Unit
*A*	Non-flying stages (larvae and pupae stages)	Individuals
*M*	Wild Males	Individuals
*F*_*W*_	Once-mated females with fertilized eggs	Individuals
*F*_*S*_	Once-mated females with non-fertilized eggs	Individuals
*F*_*SW*_	Double-mated females with non-fertilized then fertilized eggs	Individuals
*F*_*SW*_	Double-mated females with fertilized then non-fertilized eggs	Individuals
*F*_*WW*_	Double-mated females with fertilized eggs	Individuals
*F*_*SS*_	Double-mated females with non-fertilized eggs	Individuals
*M*_*S*_	Sterile Males	Individuals
*K*	Larvae/pupae mean carrying capacity	Individuals
*b*_*W*_	Mean number of viable/hatched eggs laid by female *F*_*W*_ per day	days^−1^
*b*_*WW*_	Mean number of viable/hatched eggs laid by female *F*_*WW*_ per day	days^−1^
*b*_*WS*_	Mean number of viable/hatched eggs laid by female *F*_*WS*_ per day	days^−1^
*b*_*SW*_	Mean number of viable/hatched eggs laid by female *F*_*SW*_ per day	days^−1^
*μ*_*A*_	Mortality rate of non-flying stages	days^−1^
*ν*_*A*_	Maturation rate from the non-flying stage to flying stages	days^−1^
*r*	Sex ratio	-
*μ*_*M*_	Mortality rate of wild males	day^−1^
*μ*_*F*_	Mortality rate of females mated once either with a wild or a sterile male, and double-mated females with sterile males only	day^−1^
*μ*_*F*,*WW*_	Mortality rate of females mated twice with a wild male	day^−1^
*μ*_*F*,*WS*_	Mortality rate of females mated with a wild male and then a sterile male	day^−1^
*μ*_*F*,*SW*_	Mortality rate of females mated with a sterile male and then a wild male	day^−1^
*δ*	Re-mating rate for females *F*_*W*_	day^−1^
*δ*_*S*_	Re-mating rate for sterile females *F*_*S*_	day^−1^
*μ*_*S*_	Mortality rate of sterile male	day^−1^
Λ	Sterile male release rate	individuals × days^−1^
*γ*	Competitivity parameter	-
*ε*	Proportion of fertile sperm—residual fertility	-

All linear terms represent either transfer rates, like *ν*_*A*_ and δ_*_, from one compartment to another, or death rates, *μ*_*A*_, *μ*_*M*_, *μ*_*F*_, * and *μ*_*S*_: see Table 1, page 6.

Host fruit affect the rate and duration of development, in this study we consider values from stone fruits when available, as peaches, nectarines, and apricots are the most common fruits in Corsica, together with clementines. Peach [53] and nectarine [54] appear to be very suitable fruits for the development of *C. capitata*, while clementine is less favorable for immature development [53] (see Tables 1 and 2 for the parameters used and their value). Developmental data also vary with season (temperature and fruit phenology), but those variables are not included in our model.

**Table 2:** *C. capitata* entomological parameter values used in this model (literature selected for demographic parameters were studies using host fruits rather than artificial diet, and field studies when available). The parameters values for *δ* and *δ*_*S*_ are given below in Table 3.

Parameter	Value used in the model(s)	Based on values from the literature	
		Wild Males and Females (virgin or mated with Wild males)	Sterile males (Tsl-Vienna 8 GSS)
Number of viable eggs (per day)	*b* = 12.13 day^−1^	Average values of 13 eggs per day with an egg eclosion rate of 92% for flies grown in peach [53].	(no difference)
Maturation rate from non-flying stage to flying stage	*ν*_*A*_ = 0.020 day^−1^ day^−1^	Average of 25 days from eggs to adult emergence in peach (at 25°C) and being sexually mature [53]	N/A
Non-flying stages Mortality rate	*μ*_*A*_ = 0.0227*day*^−1^	Average survival of 82.5% eggs to adult on peach (at 25°C) [53]	N/A
Average sex ratio	*r* = 0.53	Based on development on peach [53]	N/A
Adult death rate	*μ*_*F*_ = 1/42.66 day^−1^ *μ*_*M*_ = 1/50.33 day^−1^ *μ*_*S*_ = 0.23 day^−1^	Males: average lifespan of 50 days for flies grown in peach (at 25°C) [53]. Females: average lifespan of 43 days for flies grown in peach (at 25°C) [53].	Up to 50% sterile males survived over 3 days [65]; 90% of the sterile males released (exposed or not to GRO) were recaptured within 5 days [66]
Competitiveness (without GRO treatment)	*γ* = 0.6129	N/A	RSI = 0.34 ± 0.004 [66]
Competitiveness (with GRO treatment)	*γ* = 2.03	N/A	RSI = 0.67 [66]

We take into consideration male and female’s multiple mating capacity (re-mating). This parameter has been repeatedly studied in the laboratory with various estimates. However, analysis of field-sampled females progeny showed less than 28% [35] or 50% [34] multiple mating. It is thus possible to roughly estimate a percentage of female daily re-mating proportion: in average, between 7% and 12%.

This is represented, in our model, by the parameters *δ* and *δ*_*S*_, where 1/*δ* and 1/*δ*_*S*_ represent respectively the average refractory periods for the female *F*_*W*_, and the female *F*_*S*_. In general, some studies (for example [55]) showed that females *F*_*S*_ have a tendency to re-mate more often. Thus, we will assume *δ*_*S*_ ≥ *δ* ≥ 0. When a female *F*_*W*_ re-mates, then she re-mates with either a wild male or a sterile male to enter either the compartment *F*_*WW*_ at the rate $\delta \frac{M+\varepsilon \gamma M_{S}}{M+\gamma M_{S}}$ or the compartment *F*_*WS*_ at the rate $\delta \frac{\left(1-\varepsilon \right)\gamma M_{S}}{M+\gamma M_{S}}$. This is similar for a sterile female that can re-mate with a wild male and thus enter in the compartment *F*_*SW*_ at the rate $\delta_{S}\frac{M+\varepsilon \gamma M_{S}}{M+\gamma M_{S}}$.

The addition of ginger root oil (GRO) to sterile adult males is now a common process that increases the sterile males competitiveness. It has also been showed to reduce re-mating tendency, leading to similar percentages of re-mating whether females are mated first to wild or sterile males [49]. In this study we analyse the effect of GRO treatment on the release ratios.

The released number of sterile males may vary in time. Thus, we consider a release rate *u*(*t*) ≥ 0. However, for sake of simplicity and in order to go as far as possible in the qualitative analysis, we will mainly consider the constant and continuous release case, *u*(*t*) ≡ Λ. However, it is also possible to consider periodic impulsive releases, i.e.

$$u\left(t\right)=\tau \Lambda_{per}\sum_{n\in N}\delta_{t_{S}+n\tau }\left(t\right),$$

where *τ* is the given time between two consecutive releases, like 3 or 7 days, *t*_*S*_ the starting-time of the SIT treatment, and *δ* is the Dirac function.

The compartmental diagram in Fig 1, page 7, can be translated into the following mathematical model

$$\begin{array}{c} \left\{ \begin{array}{c} \frac{dA}{dt}=(b_{W}F_{W}+b_{WW}F_{WW}+b_{SW}F_{SW}+b_{WS}F_{WS})(1-\frac{A}{K})-(\nu_{A}+\mu_{A})A,\\ \frac{dM}{dt}=(1-r)\nu_{A}A-\mu_{M}M,\\ \frac{dF_{W}}{dt}=r\nu_{A}\frac{M+\varepsilon \gamma M_{S}}{M+\gamma M_{S}}A-(\delta +\mu_{F})F_{W},\\ \frac{dF_{S}}{dt}=r\nu_{A}\frac{\left(1-\varepsilon \right)\gamma M_{S}}{M+\gamma M_{S}}A-(\delta_{S}+\mu_{F})F_{S},\\ \frac{dF_{WW}}{dt}=\delta \frac{M+\varepsilon \gamma M_{S}}{M+\gamma M_{S}}F_{W}-\mu_{F,WW}F_{WW},\\ \frac{dF_{WS}}{dt}=\delta \frac{\left(1-\varepsilon \right)\gamma M_{S}}{M+\gamma M_{S}}F_{W}-\mu_{F,WS}F_{WS},\\ \frac{dF_{SW}}{dt}=\delta_{S}\frac{M+\varepsilon \gamma M_{S}}{M+\gamma M_{S}}F_{S}-\mu_{F,SW}F_{SW},\\ \frac{dF_{SS}}{dt}=\delta_{S}\frac{\left(1-\varepsilon \right)\gamma M_{S}}{M+\gamma M_{S}}F_{S}-\mu_{F}F_{SS},\\ \frac{dM_{S}}{dt}=\Lambda -\mu_{S}M_{S}, \end{array}\right. \end{array}$$ (1)

with non-negative initial conditions. Assuming *t* large enough, we may assume that *M*_*S*_ has reached its equilibrium, $M_{S}\left(0\right)=M_{S}^{*}=\frac{\Lambda }{\mu_{S}}$. In addition, the *F*_*SS*_-Eq (1)_8_ being not involved in the dynamics of the double-mating model (1), studying system (1) is equivalent to study the following system

$$\begin{array}{c} \left\{ \begin{array}{c} \frac{dA}{dt}=(b_{W}F_{W}+b_{WW}F_{WW}+b_{SW}F_{SW}+b_{WS}F_{WS})(1-\frac{A}{K})-(\nu_{A}+\mu_{A})A,\\ \frac{dM}{dt}=(1-r)\nu_{A}A-\mu_{M}M,\\ \frac{dF_{W}}{dt}=r\nu_{A}\frac{M+\varepsilon \gamma M_{S}^{*}}{M+\gamma M_{S}^{*}}A-(\delta +\mu_{F})F_{W},\\ \frac{dF_{S}}{dt}=r\nu_{A}\frac{\left(1-\varepsilon \right)\gamma M_{S}^{*}}{M+\gamma M_{S}^{*}}A-(\delta_{S}+\mu_{F})F_{S},\\ \frac{dF_{WW}}{dt}=\delta \frac{M+\varepsilon \gamma M_{S}^{*}}{M+\gamma M_{S}^{*}}F_{W}-\mu_{F,WW}F_{WW},\\ \frac{dF_{WS}}{dt}=\delta \frac{\left(1-\varepsilon \right)\gamma M_{S}^{*}}{M+\gamma M_{S}^{*}}F_{W}-\mu_{F,WS}F_{WS},\\ \frac{dF_{SW}}{dt}=\delta_{S}\frac{M+\varepsilon \gamma M_{S}^{*}}{M+\gamma M_{S}^{*}}F_{S}-\mu_{F,SW}F_{SW}. \end{array}\right. \end{array}$$ (2)

The following lemmas show that system (2) is mathematically and biologically well posed: it remains positive and bounded.

**Lemma 1** *Let M_S_ be a non-negative, piecewise continuous function on* $R_{+}$ *and assume non-negative initial data. The solution to the Cauchy problem associated with* (2) *exists on* $R_{+}^{7}$, *is unique, continuous and piecewise continuously differentiable. This solution is also forward-bounded and remains non-negative*.

It is also straightforward to show that the set $E_{7}≔\left\{A\leq K\right\}\subset R_{+}^{7}$ is forward invariant for system (2), and any trajectory enters it in finite time.

We will derive several theoretical results that help us to understand the dynamics of our SIT system. In particular, we derive a necessary condition for the residual fertility, *ε*, to hold in order to be able to control a fruit fly population.

Without sterile male releases, we recover from any of system (1) or system (2) the model of the dynamics of the natural/wild pest/vector population

$$\begin{array}{c} \left\{ \begin{array}{c} \frac{dA}{dt}=(b_{W}F_{W}+b_{WW}F_{WW})(1-\frac{A}{K})-(\nu_{A}+\mu_{A})A\\ \frac{dM}{dt}=(1-r)\nu_{A}A-\mu_{M}M,\\ \frac{dF_{W}}{dt}=r\nu_{A}A-(\delta +\mu_{F})F_{W},\\ \frac{dF_{WW}}{dt}=\delta F_{W}-\mu_{F,WW}F_{WW}. \end{array}\right. \end{array}$$ (3)

We define the basic offspring number related to model (3) as follows

$$\begin{array}{c} \begin{array}{cc} R & =R\left(\delta \right)=\frac{b_{W}r\nu_{A}}{\left(\nu_{A}+\mu_{A})(\mu_{F}+\delta \right)}+\frac{\delta }{\left(\mu_{F}+\delta \right)}\frac{b_{WW}r\nu_{A}}{\mu_{F,WW}(\nu_{A}+\mu_{A})}\\ & =N\left(\delta \right)\times (1+\frac{b_{WW}}{b_{W}}\frac{\delta }{\mu_{F,WW}}), \end{array} \end{array}$$ (4)

where

$$\begin{array}{c} N=N\left(\delta \right)=\frac{br\nu_{A}}{\left(\nu_{A}+\mu_{A})(\mu_{F}+\delta \right)}, \end{array}$$ (5)

**Remark 1** *The parameter* R *represents the average number of (female) offspring a single-mated female and a double-mated female can produce during their life time. The parameter* N *represents the average number of (female) offspring a single-mated female can produce during her life time. It is interesting to notice that* N≤R , *so that* N>1 *implies* R>1, *and* R<1 *implies* N<1.

Through straightforward calculations and using the theory of monotone cooperative system [56], we show the following result:

**Theorem 1** ([57]) *System* (3) *defines a forward dynamical system in* $E_{4}≔\left\{A\leq K\right\}\subset R_{+}^{4}$. *In addition*

- *if* R<1 , *then* $0_{R^{4}}={\left(0,0,0,0\right)}^{T}$ *is globally asymptotically stable on* $E_{4}$.
- *if* R>1 , *then a positive equilibrium* **E** *exists where*
  $$\begin{array}{c} A_{0}^{*}=(1-\frac{1}{R})K, \end{array}$$ (6)
  $$\begin{array}{c} M_{0}^{*}=\frac{\left(1-r\right)\nu_{A}}{\mu_{M}}(1-\frac{1}{R})K, \end{array}$$ (7)
  $$\begin{array}{c} F_{0,W}^{*}=\frac{r\nu_{A}}{\delta +\mu_{F}}(1-\frac{1}{R})K, \end{array}$$ (8)
  $$\begin{array}{c} F_{0,WW}^{*}=\frac{\delta }{\mu_{F,WW}}\frac{r\nu_{A}}{\delta +\mu_{F}}(1-\frac{1}{R})K, \end{array}$$ (9)
  *Furthermore*, **E** *is globally asymptotically stable on* $E_{4}\backslash \{(0,M,0,0):M\geq 0\}$.

**Proof**: see S1 Text, Sec. S1.

For the rest of the paper, we assume that R>1.

Once sterile males are released, i.e. Λ > 0, it is expected that SIT induces a strong Allee effect [58] by reducing mate finding probabilities in such a way that a population level extinction threshold exists and such that, for system (2), the steady state $0_{R^{7}}$ is Locally Asymptotically Stable (LAS). This effect is particularly useful when the pest population is either (very) small or invading the domain, because it avoids the settlement of a wild population. When the wild population is large, then it is necessary to consider a long term SIT strategy using first massive releases, and then small releases, as explained in [20, 58]. Indeed, massive releases will drive the pest population into a subset that belongs to the basin of attraction of $0_{R^{7}}$, related to a given size for the small releases, such that we can switch from massive to small releases to keep the pest population as small as needed and also to drive it slowly, but surely, towards elimination, i.e. the steady state $0_{R^{7}}$ is Locally Asymptotically Stable. In contrast, once SIT is used, it has to be maintained: if, for any reason, SIT is stopped, then the Allee effect is lost and the wild population will rise again.

To ensure that a strong Allee effect exists when sterile males are released, we show the following result related to the residual fertility, *ε*:

**Lemma 2** *When ε* ≤ *ε*_max_, *where ε*_max_ =

$$\begin{array}{c} \frac{2}{N(\delta )}\frac{1}{\left(1+\frac{\delta_{S}}{\mu_{F,SW}}\frac{b_{SW}}{b_{W}}\frac{\delta +\mu_{F}}{\delta_{S}+\mu_{F}}+\frac{\delta }{\mu_{F,WS}}\frac{b_{W,S}}{b_{W}})(\sqrt{1+\frac{4}{N(\delta )}\frac{1}{b_{W}}\frac{\delta (\frac{b_{WW}}{\mu_{F,WW}}-\frac{b_{W,S}}{\mu_{F,WS}})-\delta_{S}\frac{b_{SW}}{\mu_{F,SW}}\frac{\delta +\mu_{F}}{\delta_{S}+\mu_{F}}}{{\left(1+\frac{\delta_{S}}{\mu_{F,SW}}\frac{b_{SW}}{b_{W}}\frac{\delta +\mu_{F}}{\delta_{S}+\mu_{F}}+\frac{\delta }{\mu_{F,WS}}\frac{b_{WS}}{b_{W}}\right)}^{2}}}+1\right)}, \end{array}$$ (10)

*then* $0_{R^{7}}$ *is always Locally Asymptotically Stable (LAS) for system* (2). *It is unstable, otherwise*.

**Proof**: see S1 Text, Sec. S2.

We deduce that if the residual fertility is too large, i.e. *ε* > *ε*_max_, there is no strong Allee effect, whatever the size of sterile male releases. The upper bound for the residual fertility, *ε*_max_, given in (10), is particularly interesting, because it does not only depend on re-mating parameters *δ*, *δ*_*S*_, but also on the (hatching) eggs deposit rates and the death rates for each type of once- and double-mated Females, *F*_*W*_, *F*_*WS*_, *F*_*SW*_, and *F*_*W*,*W*_.

**Remark 2** *Without remating, i.e. δ*_*S*_ = *δ* = 0, *we recover the condition* εR(0)≤1, *as obtained in* [20], *for instance*.

**Remark 3** *According to Theorem 2, provided that ε is sufficiently small, it is possible to reach (asymptotically) elimination when* Λ *is large enough*.

In formula (10), the term

$$\begin{array}{c} F\left(\delta ,\delta_{S}\right)=\delta (\frac{b_{WW}}{\mu_{F,WW}}-\frac{b_{WS}}{\mu_{F,WS}})-\delta_{S}\frac{b_{SW}}{\mu_{F,SW}}\frac{\delta +\mu_{F}}{\delta_{S}+\mu_{F}} \end{array}$$ (11)

is particularly interesting because, depending on the previous parameters, it can be either negative, or non-negative. Thus, when F<0, then re-mating is reducing the impact of SIT, while when F≥0, re-mating is neutral or beneficial for SIT. For instance, if *δ* = *δ*_*S*_ = 0, then F=0. If *δ*_*S*_ = *δ* (equal re-mating), then

$$\begin{array}{c} F\left(\delta ,\delta \right)=\frac{\delta }{\mu_{F}}\frac{b_{WW}-b_{WS}-b_{SW}}{b_{W}}. \end{array}$$ (12)

Hence, if *b*_*WW*_ = *b*_*WS*_ + *b*_*SW*_ then F=0. Last but not least, the worst case: *δ* = 0 and *δ*_*S*_ > 0, meaning that *F*_*S*_ females re-mate but not *F*_*W*_ females, then

$$\begin{array}{c} F\left(\delta ,\delta_{S}\right)=-\delta_{S}\frac{b_{S,W}}{\mu_{F,SW}}\frac{\mu_{F}}{\delta_{S}+\mu_{F}}<0. \end{array}$$ (13)

In that case, re-mating *F*_*S*_ females only, will considerably and negatively impact SIT. We will illustrate the different cases in Sec. 2.1, page 17.

In the following proposition, we show all possible dynamics for system (2), thanks to the sterile male release rate, Λ. We also show the existence of a release rate threshold, $\Lambda_{cont,\varepsilon }^{crit,*}$ above which, elimination is possible:

**Proposition 1** *Assume* R>1 , *then the following results hold true for system* (2):

- *Assume ε* > *ε*_max_, *then there always exists one positive steady state* E* > > 0, *whatever the sterile male release rate*.
- *Assume ε* = *ε*_max_, *then, setting* $\gamma \Lambda_{cont,\varepsilon }^{crit,*}=\frac{\mu_{S}}{\frac{1}{\varepsilon_{\text{max}}R\left(\delta \right)}+\varepsilon_{\text{max}}+2\sqrt{\frac{1}{R(\delta )}}}M_{0}^{*}$, *we deduce that*
  - *If* $\Lambda \geq \Lambda_{cont,\varepsilon }^{crit,*}$, *there is no positive steady state*.
  - *If* $0\leq \Lambda <\Lambda_{cont,\varepsilon }^{crit,*}$, *there is one positive steady state* $0_{R^{7}}<{\text{E}}_{*}$.
- *Assume* 0 ≤ *ε* < *ε*_max_, *then, there exists* $\Lambda_{cont,\varepsilon }^{crit}>0$ *such that*
  - *If* $\Lambda >\Lambda_{cont,\varepsilon }^{crit}$, *there is no positive steady state*.
  - *If* $\Lambda =\Lambda_{cont,\varepsilon }^{crit,}$, *there is one positive steady state* ${\text{E}}_{\varepsilon }^{*}$.
  - *If* $0<\Lambda <\Lambda_{cont,\varepsilon }^{crit}$, *then there are two positive steady states* **E**_*ε*,−_ *and* **E**_*ε*,+_, *such that*
    $$0_{R^{7}}<{\text{E}}_{\varepsilon ,-}^{*}<{\text{E}}_{\varepsilon ,+}^{*}.$$

**Proof**: see S1 Text, Sec. S3.

**Remark 5** *Obtaining theoretically the stability properties of the positive equilibria*, $E_{\varepsilon ,-}^{*}$ *and* $E_{\varepsilon ,+}^{*}$, *when they exist, is not easy considering the complexity of the system. The numerical simulations indicate that* $E_{\varepsilon ,-}^{*}$ *is unstable while* $E_{\varepsilon ,+}^{*}$ *is asymptotically stable, and that the equilibria are the only invariant set of the system on* $R_{7}^{+}$.

In general, it is not possible to derive an explicit formula for $\Lambda_{cont,\varepsilon }^{crit}$, except when *δ* = *δ*_*S*_ = 0, but it can be estimated numerically.

However, *ε* ≤ *ε*_max_ ensures elimination only if the wild population is sufficiently small. For practical applications, and, in particular, to ensure that elimination is still possible when the wild population is large, we need to show that the steady-state $0_{R^{7}}$ is Globally Asymptotically Stable (GAS).

**Theorem 2** *Assume ε* < *ε*_max_ *and* $\Lambda >\Lambda_{cont,\varepsilon }^{crit}$, *then* $0_{R^{7}}$ *is GAS for system* (2).

**Proof**: see S1 Text, Sec. S4.

**Remark 6** *According to Theorem 2, provided that ε is sufficiently small, it is possible to reach (asymptotically) elimination when* Λ *is large enough*.

From the previous results, we deduce that the residual fertility, *ε*, is an essential parameter to take into account when designing SIT programs. As *ε* increases, the SIT becomes less effective and, eventually, fails to reduce the wild population.

The precision and homogeneity of the sterilization step is very important. Although the irradiation process is very well mastered, the level of residual fertility will depend on the homogeneity of the dose delivered to the fly pupae, which may be affected by container volume, irradiation equipment and source. The classical recommendation is to have the lowest residual fertility possible. Here we provide, for the first time, insights on the impact of residual fertility on the release success.

Technical and production improvements in medfly SIT have been obtained using genetic sex strains [59] in which males also have a naturally reduced fertility level (49.29%). The residual fertility of the VIENNA-8 GSS strain was reported to 0.84% ± 0.08% (0.6% ± 0.13%) at 100*Gy* (120*Gy*) [59, Table 3], on average. However, a higher residual fertility was observed for a similar strain (VIENNA-8 GSS strain with a Valencian background) irradiated at the same dose but with an electron beam accelerator [6], i.e.1.13% ± 0.55% for 100Gy.

Another important aspect is the re-mating of females: having good knowledge of this phenomenon can significantly change the constraint on the residual fertility, *ε*. We will illustrate the impact of re-mating in the numerical section.

However, it is important to have in mind that targeted crops may also affect the effectiveness of a SIT program. Indeed, Ceratitis capitata’s basic reproduction numbers depends on the main fruit hosts and, thus, can take a large range of values. For instance, according to [54], estimates of $R_{0}$ on deciduous fruits, like nectarine (or plum), yield $R_{0}\approx 227.28\pm 89.1$ ($R_{0}\approx 276.59\pm 54.15$), at 25°C, whereas $R_{0}\approx 27.05$ on citrus, at 24°C [60]. Clearly the constraint on *ε* will depend on the targeted host crop: for instance, a residual fertility of 2% might be acceptable for citrus but not necessarily acceptable for nectarine or plum: compare 1/27.05 ≈ 0.037 and 1/227.05 ≈ 0.0044. Section 5, where we apply our results to a real crop, we will consider medfly data related to peach, obtained in Tunisia [53], as Tunisia and Corsica show similarities from the agricultural context point of view. While continuous releases are very convenient from the theoretical point of view, it is more realistic to consider periodic instantaneous (or pulsed) releases.

### 1.1 Periodic pulsed SIT releases

We assume now that sterile males are released periodically, every *τ* days. Assuming each release as an instantaneous discrete or pulsed event, this situation can be modeled using a semi-discrete approach, like in [20, 26, 58]. Thus, system (1) becomes the following impulsive differential system

$$\begin{array}{c} \left\{ \begin{array}{c} \frac{dA}{dt}=(b_{W}F_{W}+b_{WW}F_{WW}+b_{SW}F_{SW}+b_{WS}F_{WS})(1-\frac{A}{K})-(\nu_{A}+\mu_{A})A,\\ \frac{dM}{dt}=(1-r)\nu_{A}A-\mu_{M}M,\\ \frac{dF_{W}}{dt}=r\nu_{A}\frac{M+\varepsilon \gamma M_{S}}{M+\gamma M_{S}}A-(\delta +\mu_{F})F_{W},\\ \frac{dF_{S}}{dt}=r\nu_{A}\frac{\left(1-\varepsilon \right)M_{S}}{M+\gamma M_{S}}A-(\delta_{S}+\mu_{F})F_{S},\\ \frac{dF_{WW}}{dt}=\delta \frac{M+\varepsilon \gamma M_{S}}{M+\gamma M_{S}}F_{W}-\mu_{F,WW}F_{WW},\\ \frac{dF_{WS}}{dt}=\delta \frac{\left(1-\varepsilon \right)M_{S}}{M+\gamma M_{S}}F_{W}-\mu_{F,WS}F_{WS},\\ \frac{dF_{SW}}{dt}=\delta_{S}\frac{M+\varepsilon \gamma M_{S}}{M+\gamma M_{S}}F_{S}-\mu_{F,SW}F_{SW},\\ \frac{dF_{SS}}{dt}=\delta_{S}\frac{\left(1-\varepsilon \right)\gamma M_{S}}{M+\gamma M_{S}}F_{S}-\mu_{F}F_{SS},\\ \frac{dM_{S}}{dt}=-\mu_{S}M_{S}. \end{array}\right. \end{array}$$ (14)

with the discrete sterile male releases “events” starting at time *t*_*S*_ ≥ 0

$$\begin{array}{c} \left\{ \begin{array}{c} A\left(t_{S}+n\tau_{+}\right)=A\left(t_{S}+n\tau \right),\\ M\left(t_{S}+n\tau_{+}\right)=M\left(t_{S}+n\tau \right),\\ F_{W}\left(t_{S}+n\tau_{+}\right)=F_{W}\left(t_{S}+n\tau \right),\\ F_{S}\left(t_{S}+n\tau_{+}\right)=F_{S}\left(t_{S}+n\tau \right),\\ F_{WW}\left(t_{S}+n\tau_{+}\right)=F_{WW}\left(t_{S}+n\tau \right),\\ F_{WS}\left(t_{S}+n\tau_{+}\right)=F_{WS}\left(t_{S}+n\tau \right),\\ F_{SW}\left(t_{S}+n\tau_{+}\right)=F_{SW}\left(t_{S}+n\tau \right),\\ F_{SS}\left(t_{S}+n\tau_{+}\right)=F_{SS}\left(t_{S}+n\tau \right),\\ M_{S}\left(t_{S}+n\tau_{+}\right)=M_{S}\left(t_{S}+n\tau \right)+\tau \Lambda_{per},\;n=0,1,2\ldots , \end{array}\right. \end{array}$$ (15)

and the following non-negative initial conditions

$$\begin{array}{c} \begin{array}{c} 0\leq A\left(0\right)=A_{0},\;0\leq M\left(0\right)=M_{0},\;0\leq F_{W}\left(0\right)=F_{W,0},\;F_{S}\left(0\right)=0,\;F_{SS}\left(0\right)=0,\\ 0\leq F_{WW}\left(0\right)=F_{WW,0},\;0\leq F_{WS}\left(0\right)=0,\;0\leq F_{SW}\left(0\right)=0,\;M_{S}\left(0\right)=0. \end{array} \end{array}$$ (16)

The right-hand side of system (14) and (15) is locally lipschitz continuous on $R^{9}$. Thus, using a classic existence theorem [61, Theorem 1.1, p. 3], there exist *β* > 0 and a unique solution defined from $\left(0,\beta \right)\to R^{9}$ for system (14), (15) and (16).

Thanks to Eq (14)_9_, with the pulsed event defined in Eq (15)_9_, it is straightforward to show that, as *t* → + ∞, *M*_*S*_ converges toward the periodic solution

$$M_{T,per}\left(t\right)=\frac{\tau \Lambda_{per}}{1-e^{-\mu_{S}\tau }}e^{-\mu_{S}\left(t-\left\lfloor t/\tau \right\rfloor \tau \right)},$$

such that system (14) and (15) can be reduced to

$$\begin{array}{c} \left\{ \begin{array}{c} \frac{dA}{dt}=(b_{W}F_{W}+(b_{WW}-b_{WS})F_{WW}+b_{WS}F_{TT}+b_{SW}F_{SW})(1-\frac{A}{K})-(\nu_{A}+\mu_{A})A,\\ \frac{dM}{dt}=(1-r)\nu_{A}A-\mu_{M}M,\\ \frac{dF_{W}}{dt}=r\nu_{A}\frac{M+\varepsilon \gamma M_{T,per}\left(t\right)}{M+\gamma M_{T,per}\left(t\right)}A-(\delta +\mu_{F})F_{W},\\ \frac{dF_{S}}{dt}=r\nu_{A}\frac{\left(1-\varepsilon \right)M_{T,per}}{M+\gamma M_{T,per}}A-(\delta_{S}+\mu_{F})F_{S},\\ \frac{dF_{WW}}{dt}=\delta \frac{M+\varepsilon \gamma M_{T,per}}{M+\gamma M_{T,per}}F_{W}-\mu_{F,WW}F_{WW},\\ \frac{dF_{WS}}{dt}=\delta \frac{\left(1-\varepsilon \right)M_{T,per}}{M+\gamma M_{T,per}}F_{W}-\mu_{F,WS}F_{WS},\\ \frac{dF_{SW}}{dt}=\delta_{S}\frac{M+\varepsilon \gamma M_{T,per}}{M+\gamma M_{T,per}}F_{S}-\mu_{F,SW}F_{SW}. \end{array}\right. \end{array}$$ (17)

Setting

$$\left\{ \begin{array}{c} {\underline{M}}_{T}=\underset{t\in [0,\tau ]}{\text{min}}\;M_{S}^{per}\left(t\right)=\frac{\tau \Lambda_{per}}{1-e^{-\mu_{S}\tau }}e^{-\mu_{S}\tau },\\ {\bar{M}}_{T}=\underset{t\in [0,\tau ]}{\text{max}}\;M_{S}^{per}\left(t\right)=\frac{\tau \Lambda_{per}}{1-e^{-\mu_{S}\tau }}, \end{array}\right.$$

it is obvious to deduce that, for *t* sufficiently large, system (17) is lower and upper bounded by the following two monotone systems

$$\left(U\right)\{\begin{array}{c} \frac{dA}{dt}=(b_{W}F_{W}+(b_{WW}-b_{WS})F_{WW}+b_{WS}F_{TT}+b_{SW}F_{SW})(1-\frac{A}{K})-(\nu_{A}+\mu_{A})A,\\ \frac{dM}{dt}=(1-r)\nu_{A}A-\mu_{M}M,\\ \frac{dF_{W}}{dt}=r\nu_{A}\frac{M+\varepsilon \gamma {\bar{M}}_{T}}{M+\gamma {\bar{M}}_{T}}A-(\delta +\mu_{F})F_{W},\\ \frac{dF_{S}}{dt}=r\nu_{A}\frac{\left(1-\varepsilon \right){\bar{M}}_{T}}{M+\gamma {\bar{M}}_{T}}A-(\delta_{S}+\mu_{F})F_{S},\\ \frac{dF_{WW}}{dt}=\delta \frac{M+\varepsilon \gamma {\bar{M}}_{T}}{M+\gamma {\bar{M}}_{T}}F_{W}-\mu_{F,WW}F_{WW},\\ \frac{dF_{WS}}{dt}=\delta \frac{\left(1-\varepsilon \right){\bar{M}}_{T}}{M+\gamma {\bar{M}}_{T}}F_{W}-\mu_{F,WS}F_{WS},\\ \frac{dF_{SW}}{dt}=\delta_{S}\frac{M+\varepsilon \gamma {\bar{M}}_{T}}{M+\gamma {\bar{M}}_{T}}F_{S}-\mu_{F,SW}F_{SW}, \end{array}$$

$$\left(L\right)\{\begin{array}{c} \frac{dA}{dt}=(b_{W}F_{W}+(b_{WW}-b_{WS})F_{WW}+b_{WS}F_{TT}+b_{SW}F_{SW})(1-\frac{A}{K})-(\nu_{A}+\mu_{A})A,\\ \frac{dM}{dt}=(1-r)\nu_{A}A-\mu_{M}M,\\ \frac{dF_{W}}{dt}=r\nu_{A}\frac{M+\varepsilon \gamma {\underline{M}}_{T}}{M+\gamma {\underline{M}}_{T}}A-(\delta +\mu_{F})F_{W},\\ \frac{dF_{S}}{dt}=r\nu_{A}\frac{\left(1-\varepsilon \right){\underline{M}}_{T}}{M+\gamma {\underline{M}}_{T}}A-(\delta_{S}+\mu_{F})F_{S},\\ \frac{dF_{WW}}{dt}=\delta \frac{M+\varepsilon \gamma {\underline{M}}_{T}}{M+\gamma {\underline{M}}_{T}}F_{W}-\mu_{F,WW}F_{WW},\\ \frac{dF_{WS}}{dt}=\delta \frac{\left(1-\varepsilon \right){\underline{M}}_{T}}{M+\gamma {\underline{M}}_{T}}F_{W}-\mu_{F,WS}F_{WS},\\ \frac{dF_{SW}}{dt}=\delta_{S}\frac{M+\varepsilon \gamma {\underline{M}}_{T}}{M+\gamma {\underline{M}}_{T}}F_{S}-\mu_{F,SW}F_{SW}. \end{array}$$

Then, applying Proposition 1, page 10, to system (L) and system (U), we obtain

**Proposition 2** *Assume* R>1 *and ε* < *ε*_max_, *the following hold true*.

- *When* $\Lambda_{per}>\frac{e^{\mu_{S}\tau }-1}{\tau \mu_{S}}\Lambda_{cont,\varepsilon }^{crit}$ , *the trivial equilibrium* $0_{R^{7}}$ *is Globally Asymptotically Stable for system (U)*.
- *When* $0<\Lambda_{per}\leq \frac{1-e^{-\mu_{S}\tau }}{\tau \mu_{S}}\Lambda_{cont,\varepsilon }^{crit}$ , *system (L) admits one or two positive equilibria* ${\bar{E}}_{1,7D}\leq {\bar{E}}_{2,7D}$. *In addition, if the initial data of (L) is greater than or equal to E*_1,7*D*_, *then the corresponding solution is also greater than or equal to E*_1,7*D*_. *Similarly, since* $0<{\underline{M}}_{T}<{\bar{M}}_{T}\leq \frac{\Lambda_{cont,\varepsilon }^{crit}}{\mu_{S}}$, *then system (U) admits one or two positive equilibria* ${\underline{E}}_{1,7D}\leq {\underline{E}}_{2,7D}$. *Finally, the set* $\left\{{\left(A,M,F_{W},F_{S},F_{WW},F_{WS},F_{SW}\right)}^{T}\in R_{+}^{7}:\left(A,M,F_{W},F_{S},F_{WW},F_{WS},F_{SW}\right)\leq {\underline{E}}_{1,7D}\right\}$ *belongs to the basin of attraction of* $0_{R^{7}}$ *for system (U), hence by comparison, it belongs to the basin of attraction of* $0_{R^{7}}$ *for system* (17).

From Proposition 2, we can deduce the existence of a periodic critical release rate $\Lambda_{per,\varepsilon }^{crit}>0$ such that

$$\frac{1-e^{-\mu_{S}\tau }}{\tau \mu_{S}}\Lambda_{cont,\varepsilon }^{crit}\leq \Lambda_{per,\varepsilon }^{crit}\leq \frac{e^{\mu_{S}\tau }-1}{\tau \mu_{S}}\Lambda_{cont,\varepsilon }^{crit}.$$

When *τ* goes to 0, i.e. the time between 2 consecutive releases is going to 0, we have

$$\frac{(e^{\mu_{S}\tau }-1)\Lambda_{cont,\varepsilon }^{crit}}{\tau \mu_{S}}⟶\Lambda_{cont,\varepsilon }^{crit},$$

and

$$\frac{(1-e^{-\mu_{S}\tau })\Lambda_{cont,\varepsilon }^{crit}}{\tau \mu_{S}}⟶\Lambda_{cont,\varepsilon }^{crit},$$

such that we recover the result obtained for continuous releases in Proposition 1, page 10, that is

$$\Lambda_{per,\varepsilon }^{crit}{⟶}_{\tau \to 0}\Lambda_{cont,\varepsilon }^{crit}.$$

**Proposition 3** *Assume* R>1 , *ε* < *ε*_max_, *and* $0<\tau \Lambda_{per}\leq \frac{\left(1-e^{-\mu_{S}\tau }\right)}{\mu_{S}}\Lambda_{cont,\varepsilon }^{crit}$, *the set*

$$\begin{array}{cc} \Omega_{7}= & \{{\left(A,M,F_{W},F_{S},F_{WW},F_{WS},F_{SW}\right)}^{T}\in R_{+}^{7}:\\ & {\bar{E}}_{1,7D}\leq \left(A,M,F_{W},F_{S},F_{WW},F_{WS},F_{SW}\right)\leq \text{E}\} \end{array}$$

*is positively invariant by system* (17), *where* E, *the initial wild equilibrium, is defined in Theorem 1, page 9*.

Finally, using the previous result and Brouwer fixed point theorem, with comparison arguments, it is possible to show

**Theorem 3** *Assume* R>1 , *ε* < *ε*_max_, *and* $0<\tau \Lambda_{per}\leq \frac{\left(1-e^{-\mu_{S}\tau }\right)}{\mu_{S}}\Lambda_{cont,\varepsilon }^{crit}$. *Then, for each initial condition in* Ω_7_, *system* (17) *has at least one positive τ-periodic solution* **E**_*per*_ *such that* **E**_*per*_ ∈ Ω_7_.

It is not possible to find an analytical formula for $\Lambda_{per,\varepsilon }^{crit}$. However, it is possible to estimate it numerically, by solving system (14), (15) and (16), page 12, using an iterative approach. When *τ* goes to 0, meaning that the periodic releases become continuous, $\Lambda_{per,\varepsilon }^{crit}$ converges to $\Lambda_{cont,\varepsilon }^{crit}$.

## 2 Results

We now apply the previous theoretical results to the medfly, *Ceratis capitata*, parameters. We summarize in Table 2, page 16, the values estimated for some biological parameters obtained from the literature for medfly grown on peach (when available) and on sterile males from the V8 GSS strain (Vienna-8 Genetic Sexing Strain).

All computations have been done using codes developed with the software R-studio (Version 2023.06.0+421) [62], and R (version 4.2.2 (2022-10-31)) [63]. In particular, estimates of the critical periodic ratio, $\frac{\tau \Lambda_{per,\varepsilon }^{crit}}{M_{0}^{*}}$, have been obtained by solving system (14), page 12, using a nonstandard finite difference scheme (see [64] and references therein), in order to derive a fast algorithm to obtain each figure in a reasonable amount of time, on a laptop. However, for readers convenience, the R-codes [63] are also available here: https://gitlab.com/cirad-apps/residual-fertility-and-re-mating-in-sit.

In the forthcoming simulations, we express the critical thresholds, $\Lambda_{cont,\varepsilon }^{crit}$ and $\tau \Lambda_{per,\varepsilon }^{crit}$ in terms of the quantity $M_{0}^{*}$ of wild males at equilibrium, coherently with what is done in SIT programmes.

### 2.1 Parameterization and simulations

One of the recurrent difficulties in using modeling to understand biological phenomena lies in the necessity to estimate the values of the biological parameters. Most behavioral or biological studies are made in laboratory settings and under controlled conditions; they do not necessarily reflect behavior in the field. On the other hand, reports from field studies may cover only a small portion of the diversity of behaviors or physiology of wild populations. Most of the parameters values here were estimated directly or indirectly from the literature, selecting studies that brought the closest estimates to what may occur in the field rather than laboratory studies, when possible.

Since we have estimates of only part of the whole parameter set, we will consider parameters values from [53], on peach, and also use results from [42, 43, 46] for some parameters related to re-mating.

We estimate *ν*_*A*_ and *μ*_*A*_ from the data provided on peach development [53] as follows. Since $\frac{1}{\nu_{A}+\mu_{A}}=18.1$ [53, Table 2], and, the proportion of hatched eggs that become adults is 66.09% [53, Table 3], this means that $\frac{\nu_{A}}{\nu_{A}+\mu_{A}}=0.6609$, that is $\nu_{A}=\frac{0.6609}{18.1}\approx 0.0365$ day^−1^, and *μ*_*A*_ = 0.0187 day^−1^. Similarly, we deduce that *b*_*W*_ = 13.19 × 0.92 = 12.135 day^−1^ [53, Tables 1 and 3].

Little literature exists on development parameters from fruit host rather than artificial diet. Big variations may occur according to the host, citrus or clementine having lower developmental rate and longer duration but a higher adult lifespan, as compared to peach hosts [53].

The model from Plant & Cunningham [65] predicted that 50% of the released sterile males were dead after 3 days; assuming that the population size is given by $P(t)=P(0)e^{-\mu_{s}t}$ we derive that $\mu_{S}=\frac{\text{ln}\;2}{3}=0.2310$.

The estimation of the sterile male competitiveness index is based on the Relative Sterility Index (RSI), through cage experiments. Without GRO treatment, the mean RSI is low, around 0.38, but still greater than the threshold 0.2, leading to a competitiveness parameter $\gamma =\frac{RSI}{1-RSI}=0.61$ [49]. When sterile males are treated with an optimal dose of ginger root oil (GRO) [49], then the mean RSI becomes 0.67 which implies a competitive parameter *γ* = 2.03. Notice that sterile and wild males are equally competitive when RSI = 0.5, for a 1 : 1 : 1 density. The parameters values are summarized in Table 2, page 16.

Some field studies reported percentages of double mating in wild females: up to 50% [34]; less than 28% [35]; 4% to 28% [67]. Laboratory tests reported by Abraham et al [31] indicated 20% re-mating for females mated with 100Gy-irradiated males. Recently, Pogue et al have shown that female mating propensity reduces over time [68]: with an average of 75% females *C.capitata* re-mating a second time but only less than 25% performing more successive re-mating. The literature on medfly remating varies with studies and the global picture is not yet complete, as reviewed by Pérez-Staple & Abraham [69]. Here, we have chosen to estimate the parameters *δ* and *δ*_*S*_ based on the values reported from laboratory experiments by [36, Table 1]. From competing scenarios with wild and sterile males, we could estimate that the re-mating rate was equal to the proportion of re-mating, (40% when females are mated first with a wild male), divided by the mean refractory period (RFP), (i.e. 2.5 days), in such a way that we get $\delta =\frac{0.4}{2.5}=0.16$ day^−1^ for females exposed to sterile males not treated with GRO. Using the same reasoning we also derive *δ*_*S*_ with and without GRO-treatment: see Table 3.

**Table 3:** Re-mating rates with and without GRO treatment [36].

	*δ*	*δ*_*S*_
SM	0.4/2.5 = 0.16	0.64/2.02 = 0.3161
SM-GRO	0.2/2 = 0.1	0.34/1.44 = 0.2361

The lifespan of sterile males was reported not to be impacted by the GRO treatment (see [66]).

Re-mating has an important impact on females fitness: see [42, 44, 46]. In [46], single-mated females live on average 27 days, while multiple-mated females live on average 34 days, so we deduce $\mu_{WW}=\frac{27}{34}\mu_{F}$. Similarly, the average number of offspring (not the amount of hatched eggs deposit every day) is, on average, 87 for single-mated females, and 142 for double-mated female over their lifetime. Thus daily, single-mated females produces, on average, 3.22 offspring per day, and multiple-mated females produces 4, 1765 offspring per day. Thus, we deduce that the production of (hatched) eggs for a double-mated female is, on average, $b_{WW}=\frac{4,1765}{3.22}\times b_{W}$. This last result, *b*_*WW*_ > *b*_*W*_, is confirmed in [44].

For the females, *F*_*WW*_, *F*_*WS*_ and *F*_*SW*_, there is no fecundity and lifespan data available related to re-mating with the V8-strain. Only few contrasting partial data are available for *C. capitata* in [42] and [43], from which we can extrapolate some numerical values of *b*_*WS*_, *b*_*SW*_, but not for the death-rates, *μ*_*WS*_, and *μ*_*SW*_. In [42], the authors considered two genotypes: they showed that copulation order with different genotypes (irradiated or not) may influence the fitness of the double-mated females. We will consider [43], even if some data are missing. Using [43, Table 2] we derive $b_{WS}=\frac{44.2}{93.7}\times b_{W}=0.4717\times b_{W}$ and $b_{SW}=\frac{61.4}{93.7}\times b_{W}=0.6553\times b_{W}$. These are of course rough estimates. It would be preferable to have experiments estimating simultaneously the birth rates and the death rates for the different compartments. Until then, we will assume that *μ*_*WS*_ = *μ*_*SW*_ = *μ*_*F*_.

**Remark 7** *It is interesting to notice that data to estimate* *b*_*WS*_ *and b*_*SW*_ *also exist for B. dorsalis in* [70], *showing the first male sperm precedence. Indeed, if the total amount of laid eggs is (statistically) similar for F*_*WS*_ *and F*_*SW*_, *the proportion of eggs hatched is not: it is* 71.7% *for F*_*WS*_ *and* 54.9% *for F*_*SW*_. *It is important to notice that this result shows that the first sperm seems to have the precedence to the second sperms since b*_*WS*_ > *b*_*SW*_, *at least for B. dorsalis. There is no information about the death-rates, μ*_*WS*_ *and μ*_*SW*_.

From [71], for *C. capitata*, there is a tendency of second sperm precedence, at least for the first ovipositions, and then it decreases in favor of the first sperm. However, it would be more than welcome to conduct experiments, like [70], to clearly estimate the total amount of hatching eggs laid by *F*_*WS*_, *F*_*SW*_ and, also *F*_*WW*_, and also their mean lifespan, to estimate the birth rates and the death rates for these compartments.

Using Table 3, page 18, we derive Table 4, page 19, where the values for the basic offspring number are computed according to the two different sub-cases: with re-mating, R(δ), without re-mating, R(0), with and without GRO-treatment. As shown, the impact of re-mating is important on the basic offspring number: the smaller the refractory period the larger the basic offspring number. Clearly, with or without GRO-treatment, there is a need to study the mating and re-mating behaviour of females that mated either with wild males or sterile males.

**Table 4:** Basic Offspring numbers with and without GRO treatment according to Tables 2 and 3.

	R(0)	R(δ)
SM	181.4221	228.4270
SM-GRO	91.4567	138.3790

The tolerable value for residual fertility is very low according to the chosen parameters values. This would give reason to SIT implementation programs that choose a fully sterilizing dose, such as [31].

Thanks to S1 Text, Sec. 3, by solving

$$\Psi \left(M\right)\equiv (M-M_{1}^{*})(M-M_{2}^{*})(1-\frac{\mu_{M}}{\left(1-r\right)\nu_{A}K}M)=\frac{1}{R(\delta )}{\left(M+\gamma M_{S}\right)}^{2}\equiv \Phi \left(M\right),$$

we are able to derive estimates of $\frac{\Lambda_{cont,\varepsilon }^{crit}}{M_{0}^{*}}$ to highlight the issues of residual fertility and re-mating in SIT control treatment. In the forthcoming simulations we consider four cases for the re-mating rates:

(a) Assume that *δ* = 0 and *δ*_*S*_ > 0. This means that females that mated with a sterile male will always re-mate. This, would be the worst case, but it is interesting to see how this impacts the SIT treatment.
(b) No re-mating, i.e. *δ* = *δ*_*S*_ = 0: females mate only once whether the male is wild or sterile.
(c) With re-mating, such that *δ* = *δ*_*S*_ > 0. This is what we called “equal remating”, i.e. female will re-mate at the same rate, whether the male is wild or sterile.
(c) With re-mating, such that 0 < *δ* < *δ*_*S*_. This is supposed to be the “standard” case for *C. capitata*: after mating with a sterile male, a female will re-mate faster than a female that mated with a wild male.

We also consider different numerical values for *b*_*WS*_ and *b*_*SW*_ for continuous and periodic releases to illustrate the importance of the double-mated females parameters. In all simulations, SIT-treatment starts at time *t* = 100 days.

**Fig 2:**
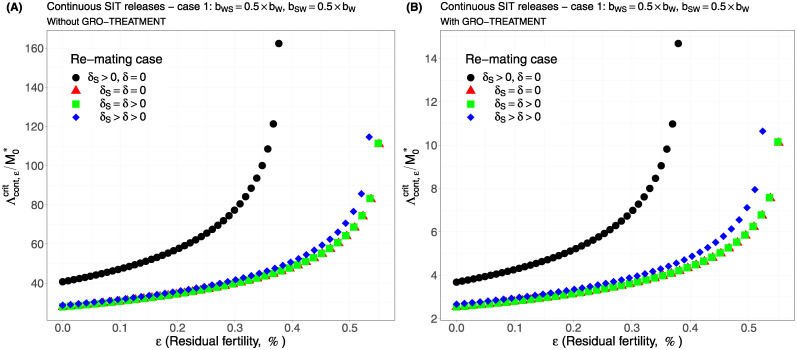
Critical ratio for continuous releases as a function of residual fertility—re-mating case 1 with *b*_*W*,*S*_ = 0.5 × *b*_*W*_, *b*_*S*,*W*_ = 0.5 × *b*_*W*_: A) without GRO-treatment. B) With GRO-treatment. Simulations with different re-mating configurations: the black bullets, with re-mating rates *δ*_*S*_ > 0 and *δ* = 0; the red triangles, the NO re-mating case, *δ*_*S*_ = *δ* = 0; the green squares, with positive and equal re-mating rates, *δ*_*S*_ = *δ* > 0; the blue diamonds, with positive re-mating rates, *δ*_*S*_ > *δ* > 0.

**Fig 3:**
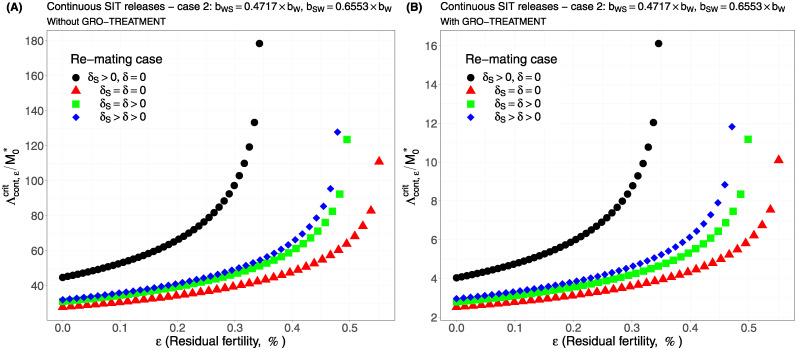
Critical ratio for continuous releases as a function of residual fertility—re-mating case 2 with *b*_*W*,*S*_ = 0.4717 × *b*_*W*_ and *b*_*SW*_ = 0.6553 × *b*_*W*_: A) without GRO-treatment. B) With GRO-treatment. Simulations with different re-mating configurations: the black bullets, with re-mating rates *δ*_*S*_ > 0 and *δ* = 0; the red triangles, the NO re-mating case, *δ*_*S*_ = *δ* = 0; the green squares, with positive and equal re-mating rates, *δ*_*S*_ = *δ* > 0; the blue diamonds, with positive re-mating rates, *δ*_*S*_ > *δ* > 0.

**Fig 4:**
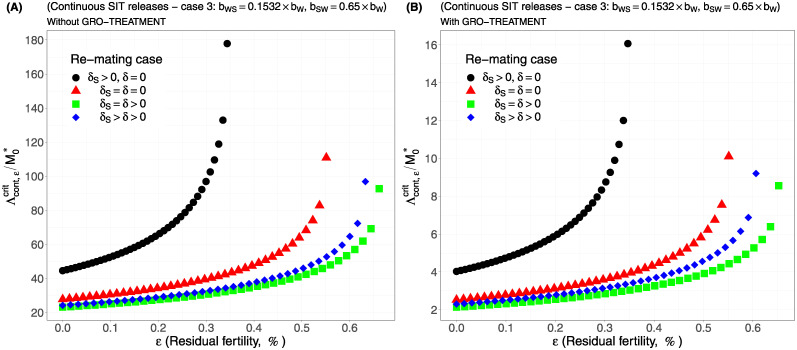
Critical ratio for continuous releases as a function of residual fertility—re-mating case 3 with *b*_*WS*_ = 0.1532 × *b*_*W*_ and *b*_*SW*_ = 0.65 × *b*_*W*_: A) without GRO-treatment. B) With GRO-treatment. Simulations with different re-mating configurations: the black bullets, with re-mating rates *δ*_*S*_ > 0 and *δ* = 0; the red triangles, the NO re-mating case, *δ*_*S*_ = *δ* = 0; the green squares, with positive and equal re-mating rates, *δ*_*S*_ = *δ* > 0; the blue diamonds, with positive re-mating rates, *δ*_*S*_ > *δ* > 0.

**Fig 5:**
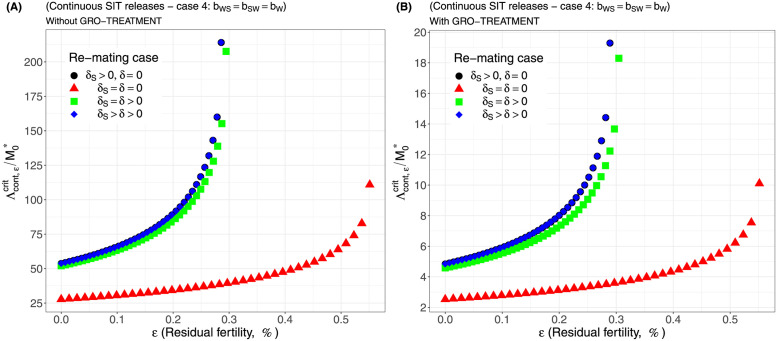
Critical ratio for continuous releases as a function of residual fertility—re-mating case 4 with *b*_*W*,*S*_ = *b*_*W*_, *b*_*S*,*W*_ = *b*_*W*_: A) without GRO-treatment. B) With GRO-treatment. Simulations with different re-mating configurations: the black bullets, with re-mating rates *δ*_*S*_ > 0 and *δ* = 0; the red triangles, the NO re-mating case, *δ*_*S*_ = *δ* = 0; the green squares, with positive and equal re-mating rates, *δ*_*S*_ = *δ* > 0; the blue diamonds, with positive re-mating rates, *δ*_*S*_ > *δ* > 0.

Case 1 Assume *b*_*W*,*S*_ = *b*_*S*,*W*_ = 0.5 × *b*_*W*_.
  Comparing Fig 2A and 2B, page 20, GRO-treatment improves drastically the ratio between the critical release rate and the amount of wild males at equilibrium, $M_{0}^{*}$. The gain in release rate is nearly 10 with the GRO treatment compared to without the GRO treatment. The larger the residual fertility, the larger the amount of sterile males to release. However, it is interesting to notice that this increase is low for *ε* < 0.25%. Of course, as expected, the worst case is the case when matings with sterile males induce a re-mating, *δ*_*S*_ > 0, while there is no re-mating when matings occur with wild male, *δ* = 0 (the blue curve). From Table 5, we see that GRO-treatment does not change much the upper bound for *ε*_*max*_, the threshold value for the residual fertility, defined in (10), page 9.
Case 2 As explained above, and following [43], we assume now that *b*_*WS*_ = 0.4717 × *b*_*W*_ and *b*_*SW*_ = 0.6553 × *b*_*W*_: see Fig 3A and 3B, page 20.
  Contrary to case 1, re-mating mainly impacts *ε*_max_: see Table 6, page 20. Indeed, when re-mating occurs, with *δ*_*S*_ > *δ* > 0, the maximal residual fertility decreases, *ε*_max_ = 0.0047, compared to case 1, where *ε*_max_ = 0.0052. However, the values for the critical release rate are similar between case 1 and case 2, at least when *ε* < 0.25%. The GRO-treatment does not change much the upper bound for *ε*_*max*_.
Case 3 This case is more or less related to the results obtained in [42]: see cases F and G in Table 2 [42], where the sterile male is not of the same genotype than the wild male. Such a case could eventually occur. Thanks to Tables 1 and 2 in [42], we assume that *b*_*WS*_ = 0.1532 × *b*_*W*_ and *b*_*SW*_ = 0.65 × *b*_*W*_: see Fig 4A and 4B, page 21. As expected, this is a case where re-mating is favorable for SIT: *ε*_max_ takes larger values when *δ* > 0: see Table 7, page 21. Note also, that in [42, Table 3], the cases E and H (the reverse cases to cases F and G) may be even more favorable for re-mating. However, there is no change in the critical release rates compared to cases 1 and 2, except for the re-mating cases where *δ*_*S*_ ≥ *δ* > 0: see Fig 4A and 4B, page 21.
Case 4 The worst case: re-mated Females *F*_*W*,*S*_ and *F*_*S*,*W*_ are always using the fertile sperm (sterile sperm is not competitive or females are able to distinguish between sterile and fertile sperm): *b*_*W*,*S*_ = *b*_*S*,*W*_ = *b*_*W*_. The results are almost similar for all re-mating cases: see Fig 5A and 5B, page 22.

**Table 5:** Numerical estimates for *ε*_max_, the threshold value for the residual fertility, with and without GRO treatment—Case 1: *b*_*W*,*S*_ = *b*_*S*,*W*_ = 0.5 × *b*_*W*_.

	*δ*_*S*_ > *δ* > 0	*δ*_*S*_ = *δ* > 0	*δ*_*S*_ > 0, *δ* = 0	*δ*_*S*_ = *δ* = 0
*ε*_max_	0.00534	0.00549	0.00376	0.00551
*ε*_max,*GRO*_	0.00524	0.00549	0.00379	0.00551

**Table 6:** Numerical estimates for *ε*_max_ with and without GRO treatment—Case 2: *b*_*WS*_ = 0.4717 × *b*_*W*_ and *b*_*SW*_ = 0.6553 × *b*_*W*_.

	*δ*_*S*_ > *δ* > 0	*δ*_*S*_ = *δ* > 0	*δ*_*S*_ > 0, *δ* = 0	*δ*_*S*_ = *δ* = 0
*ε*_max_	0.00479	0.00495	0.00343	0.00551
*ε*_max,*GRO*_	0.00471	0.00499	0.00345	0.00551

**Table 7:** Numerical estimates for *ε*_max_ with and without GRO treatment—Case 3: *b*_*WS*_ = 0.1532 × *b*_*W*_ and *b*_*SW*_ = 0.65 × *b*_*W*_.

	*δ*_*S*_ > *δ* > 0	*δ*_*S*_ = *δ* > 0	*δ*_*S*_ > 0, *δ* = 0	*δ*_*S*_ = *δ* = 0
*ε*_max_	0.00629	0.00661	0.00344	0.00551
*ε*_max,*GRO*_	0.00607	0.00652	0.00347	0.00551

We can notice that in all simulations, the gain, in the critical release ratio, is almost of factor 10 with the GRO-treatment. On the other hand there is almost no gain on *ε*_max_.

It is interesting to compare the upper bounds, *ε*_max_, given in Tables 5–8, with real values. For instance, in [59, Table 3], the sterility induced by 100GY (120GY) sterilized males was 99.42% ± 0.1 (99.72% ± 0.15), that is an average residual fertility of 0.58% (0.28%), when considering the percentage of pupae surviving from progeny of females mated by sterilized males. This is close to the upper bounds given in Tables 5 and 7, whether re-mating occurs, with *δ* > 0, or not. On the other hand, in [6], the sterility induced by 100GY sterilized males was 98.87% ± 0.55 providing an average residual fertility of 1.13%, that is *ε* = 0.0113. This value is much larger than the values given in Tables 5, 6, 7 and 8. Following our theoretical results, this means that the sterile males released under the conditions of [6] could only induce a reduction of the wild population, but not an elimination. In fact, it seems clear that estimating the residual fertility alone is not sufficient: the impact of residual fertility might depend on the values taken by *b*_*W*_, *μ*_*W*_, *b*_*WW*_, *μ*_*WW*_, *b*_*SW*_, *μ*_*SW*_, *b*_*WS*_, *μ*_*WS*_, *δ*, *δ*_*S*_, and also *γ*.

**Table 8:** Numerical estimates for *ε*_max_ with and without GRO treatment—Case 4: *b*_*W*,*S*_ = *b*_*S*,*W*_ = *b*_*W*_.

	*δ*_*S*_ > *δ* > 0	*δ*_*S*_ = *δ* > 0	*δ*_*S*_ > 0, *δ* = 0	*δ*_*S*_ = *δ* = 0
*ε*_max_	0.00285	0.00294	0.00286	0.00551
*ε*_max,*GRO*_	0.00289	0.00304	0.00290	0.00551

**Fig 6:**
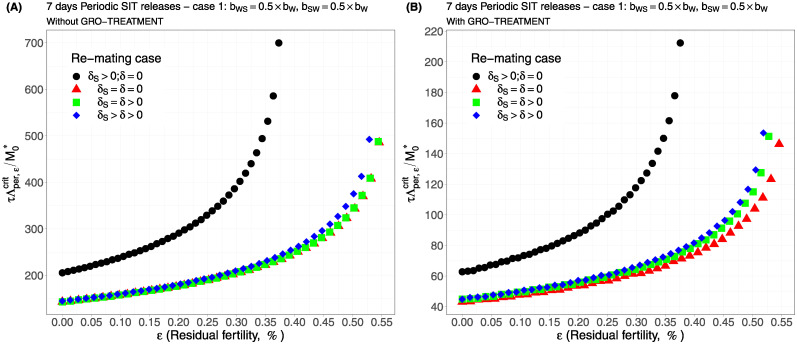
Critical ratio for weekly periodic releases as a function of residual fertility—re-mating 1 with *b*_*W*,*S*_ = 0.5 × *b*_*W*_, *b*_*S*,*W*_ = 0.5 × *b*_*W*_: A) without GRO-treatment. B) With GRO-treatment. Simulations with different re-mating configurations: the black bullets, with re-mating rates *δ*_*S*_ > 0 and *δ* = 0; the red triangles, the NO re-mating case, *δ*_*S*_ = *δ* = 0; the green squares, with positive and equal re-mating rates, *δ*_*S*_ = *δ* > 0; the blue diamonds, with positive re-mating rates, *δ*_*S*_ > *δ* > 0.

**Fig 7:**
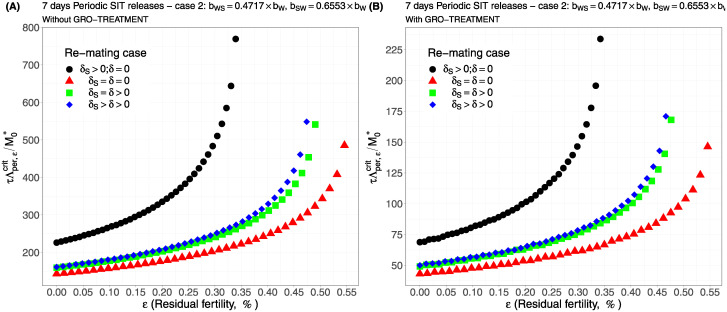
Critical ratio for weekly periodic releases as a function of residual fertility—re-mating case 2 with *b*_*W*,*S*_ = 0.4717 × *b*_*W*_ and *b*_*SW*_ = 0.6553 × *b*_*W*_: A) without GRO-treatment. B) With GRO-treatment. Simulations with different re-mating configurations: the black bullets, with re-mating rates *δ*_*S*_ > 0 and *δ* = 0; the red triangles, the NO re-mating case, *δ*_*S*_ = *δ* = 0; the green squares, with positive and equal re-mating rates, *δ*_*S*_ = *δ* > 0; the blue diamonds, with positive re-mating rates, *δ*_*S*_ > *δ* > 0.

**Fig 8:**
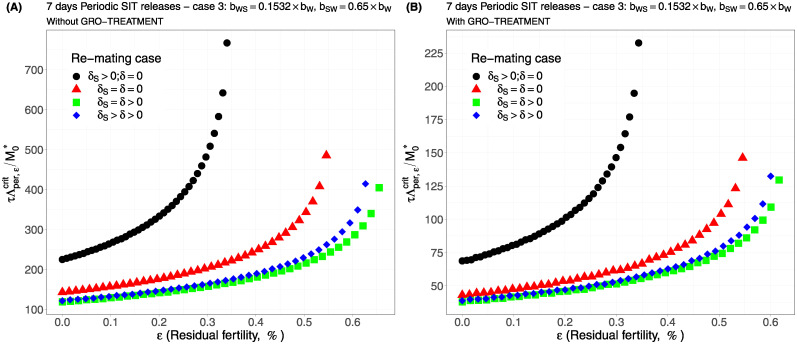
Critical ratio for weekly periodic releases as a function of residual fertility—re-mating case 3 with *b*_*WS*_ = 0.1532 × *b*_*W*_ and *b*_*SW*_ = 0.65 × *b*_*W*_: A) without GRO-treatment. B) With GRO-treatment. Simulations with different re-mating configurations: the black bullets, with re-mating rates *δ*_*S*_ > 0 and *δ* = 0; the red triangles, the NO re-mating case, *δ*_*S*_ = *δ* = 0; the green squares, with positive and equal re-mating rates, *δ*_*S*_ = *δ* > 0; the blue diamonds, with positive re-mating rates, *δ*_*S*_ > *δ* > 0.

**Fig 9:**
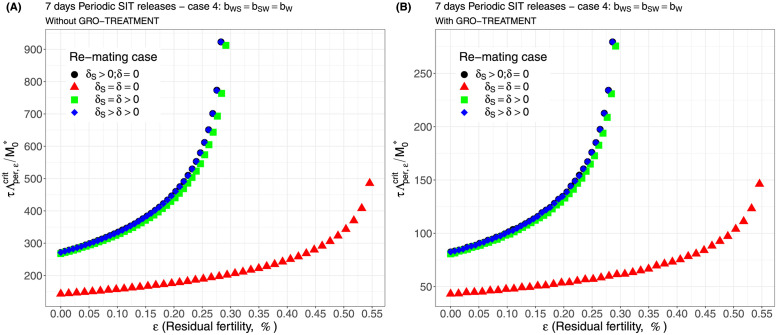
Critical ratio for weekly periodic releases as a function of residual fertility—re-mating case 4 with *b*_*W*,*S*_ = *b*_*S*,*W*_ = *b*_*W*_: A) without GRO-treatment. B) With GRO-treatment. Simulations with different re-mating configurations: the black bullets, with re-mating rates *δ*_*S*_ > 0 and *δ* = 0; the red triangles, the NO re-mating case, *δ*_*S*_ = *δ* = 0; the green squares, with positive and equal re-mating rates, *δ*_*S*_ = *δ* > 0; the blue diamonds, with positive re-mating rates, *δ*_*S*_ > *δ* > 0.

**Fig 10:**
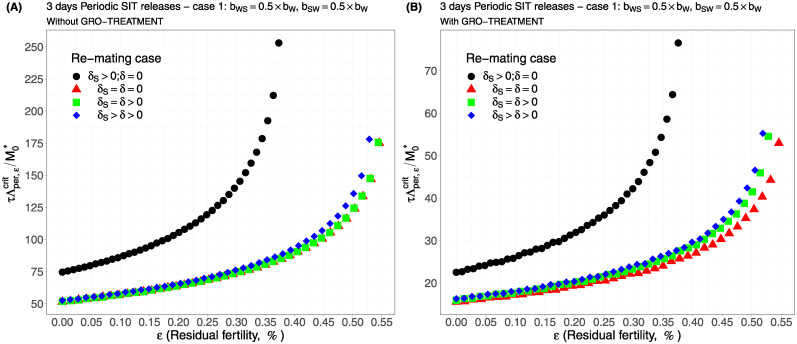
Critical ratio for periodic releases every 3 days based on residual fertility—re-mating case 1 with *b*_*W*,*S*_ = 0.5 × *b*_*W*_, *b*_*S*,*W*_ = 0.5 × *b*_*W*_: A) without GRO-treatment. B) With GRO-treatment. Simulations with different re-mating configurations: the black bullets, with re-mating rates *δ*_*S*_ > 0 and *δ* = 0; the red triangles, the NO re-mating case, *δ*_*S*_ = *δ* = 0; the green squares, with positive and equal re-mating rates, *δ*_*S*_ = *δ* > 0; the blue diamonds, with positive re-mating rates, *δ*_*S*_ > *δ* > 0.

**Fig 11:**
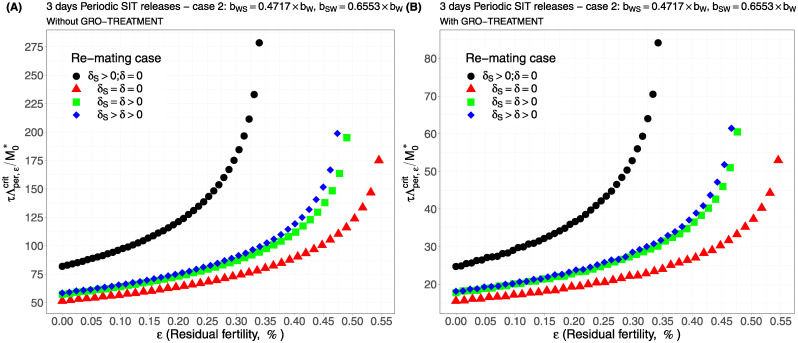
Critical ratio for periodic releases every 3 days based on residual fertility—re-mating case 2 with *b*_*W*,*S*_ = 0.4717 × *b*_*W*_ and *b*_*SW*_ = 0.6553 × *b*_*W*_: A) without GRO-treatment. B) With GRO-treatment. Simulations with different re-mating configurations: the black bullets, with re-mating rates *δ*_*S*_ > 0 and *δ* = 0; the red triangles, the NO re-mating case, *δ*_*S*_ = *δ* = 0; the green squares, with positive and equal re-mating rates, *δ*_*S*_ = *δ* > 0; the blue diamonds, with positive re-mating rates, *δ*_*S*_ > *δ* > 0.

**Fig 12:**
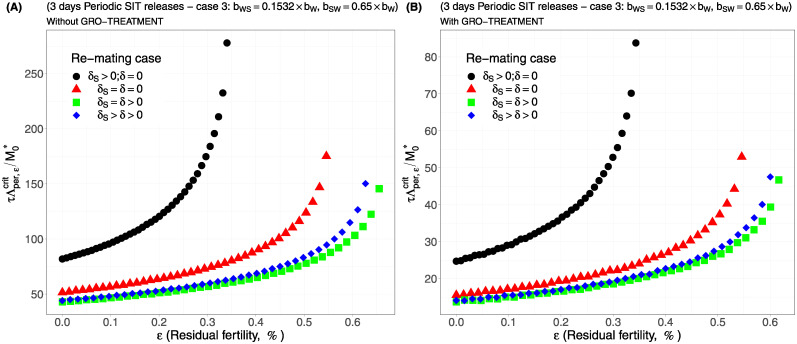
Critical ratio for periodic releases every 3 days based on residual fertility—re-mating case 3 with *b*_*WS*_ = 0.1532 × *b*_*W*_ and *b*_*SW*_ = 0.65 × *b*_*W*_: A) without GRO-treatment. B) With GRO-treatment. Simulations with different re-mating configurations: the black bullets, with re-mating rates *δ*_*S*_ > 0 and *δ* = 0; the red triangles, the NO re-mating case, *δ*_*S*_ = *δ* = 0; the green squares, with positive and equal re-mating rates, *δ*_*S*_ = *δ* > 0; the blue diamonds, with positive re-mating rates, *δ*_*S*_ > *δ* > 0.

**Fig 13:**
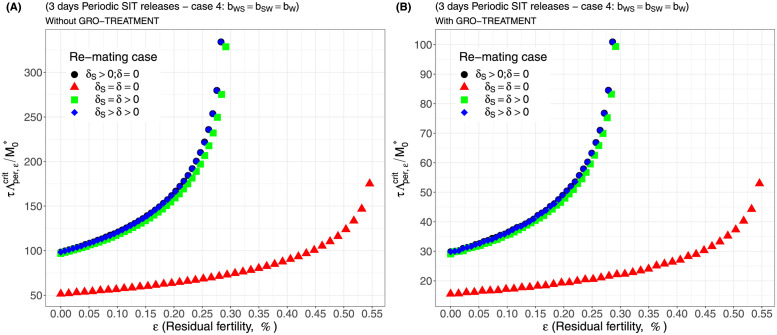
Critical ratio for periodic releases every 3 days based on residual fertility—re-mating case 4 with *b*_*W*,*S*_ = *b*_*S*,*W*_ = *b*_*W*_: A) without GRO-treatment. B) With GRO-treatment. Simulations with different re-mating configurations: the black bullets, with re-mating rates *δ*_*S*_ > 0 and *δ* = 0; the red triangles, the NO re-mating case, *δ*_*S*_ = *δ* = 0; the green squares, with positive and equal re-mating rates, *δ*_*S*_ = *δ* > 0; the blue diamonds, with positive re-mating rates, *δ*_*S*_ > *δ* > 0.

For periodic releases, we fully rely on numerical simulations of system (17), page 13, to estimate $\frac{\tau \Lambda_{\varepsilon ,per}^{crit}}{M_{0}^{*}}$, for a given period of releases, *τ*. We consider the four cases studied in the continuous release part. As expected, whatever the case, the weekly releases provide bad results, i.e. massive releases are requested: see Fig 6A and 6B, page 22, Fig 7A and 7B, page 22, Fig 8A and 8B, page 22, and Fig 9A and 9B, page 22. This is not surprising as we consider a short lifespan for sterile males. The best release period is 3 days as showed in Fig 10A and 10B, page 22, Fig 11A and 11B, page 22, Fig 12A and 12B, page 23, and Fig 13A and 13B, page 23. Again, the GRO-treatment is very helpful, and if residual fertility is low, say less than 0.3%, then the release ratios are reasonable in terms of production: see all left figures, from Fig 6A, page 22, to Fig 13A, page 23. Note also the critical release ratio for periodic releases cannot directly be deduced from the continuous critical release ratio, $\Lambda_{cont,\varepsilon }^{crit}$, by multiplying it by *τ*, the release period, because, in general, $\Lambda_{per}^{crit}>\tau \Lambda_{cont,\varepsilon }^{crit}$.

Last but not least, for periodic releases, whatever the release period, the gain in sterile male releases is only a factor 3 − 4 with the GRO treatment compared to without the GRO treatment.

**Fig 14:**
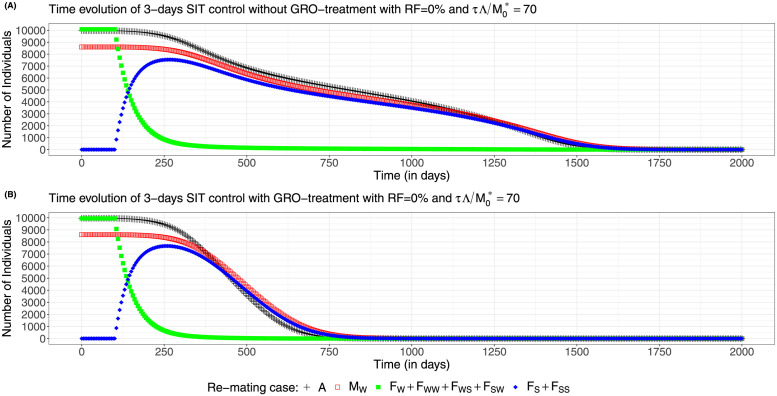
Wild population numbers evolution in time for periodic releases every 3 days and no residual fertility—re-mating case 2 with *b*_*W*,*S*_ = 0.4717 × *b*_*W*_ and *b*_*SW*_ = 0.6553 × *b*_*W*_: (A) Without GRO-treatment (B) With GRO-treatment.

**Fig 15:**
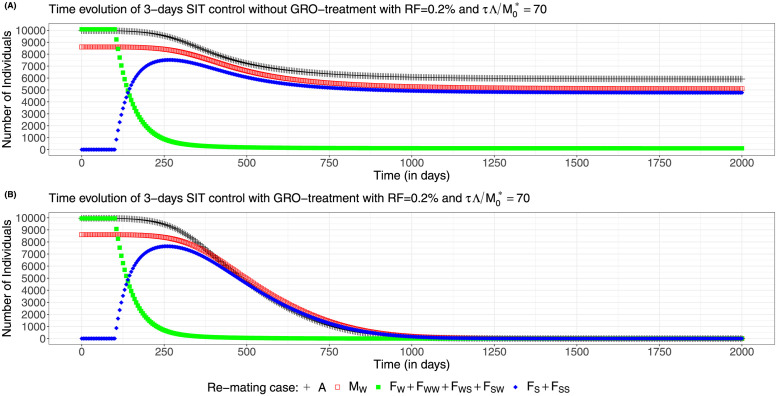
Wild population numbers evolution in time for periodic releases every 3 days and 0.2% residual fertility—re-mating case 2 with *b*_*W*,*S*_ = 0.4717 × *b*_*W*_ and *b*_*SW*_ = 0.6553 × *b*_*W*_,: (A) Without GRO-treatment (B) With GRO-treatment.

We also provide some simulations to show the dynamics of system (14), page 12, for case 2 with a 3-days SIT release strategy, for different residual fertility, 0% and 0.2%, showing the difference between no-GRO treatment and GRO-treatment: see and compare Fig 14, page 23, and Fig 15, page 24. We choose the size of the release such that $\tau \Lambda /M_{0}^{*}=70$: when *ε* = 0, thanks to Fig 11 page 22, we are above the critical values with and without GRO-treatment. In contrary, when *ε* = 0.002, without GRO-treatment, we are below the critical value, and above when GRO-treatment is used.

As expected the dynamics can be rather different: in Fig 14(A), page 23, the release ratio, 70, is just above the critical release ratio (≈ 68), that is why it takes so long to reach elimination, while in Fig 14(B), page 23, elimination is reached in less than 3 years. Even if the wild females (yellow curve) first decay rapidly, during the first 150 days after the starting date, they converge slowly to 0. In the mean time, the sterile females population, *F*_*S*_ + *F*_*SS*_ increase rapidly and then decay following the decay of the wild males population, *M*_*W*_, and the non-adult stage population, *A*. When residual fertility occurs, with *ε* = 0.002, with the same release ratio, we show in Fig 15(A), page 24, that the system converges to a new positive equilibrium, still with a large population, despite the massive release. When GRO-treatment is used, then elimination is reached in less than 3 years: see Fig 15(B), page 24. It is interesting to note, in Fig 15(A), that while the amount of fertile females is small (a few hundreds), wild males and non-adult stages are very large. This is due to the fact that the amount of sterile females is large, so that the *F*_*S*_ females can potentially, re-mate with wild males and then have offspring. That is why, elimination can only be ensured when the fertile population and also the *F*_*S*_ population have been eliminated.

Of course, once elimination is nearly reached, massive releases are no more necessary: using the strong Allee effect, we can switch to small releases, like in [58], to maintain the wild population very low and to converge slowly but surely to elimination.

Note also that the critical release ratios obtained in the simulations are more or less comparable to the ratios given in [72, Table 3].

## 3 Discussion

The overall success of an SIT program relies on (1) the field efficacy of sterile males in securing matings and sterile progeny and (2) reasonable production costs. The former is affected by several factors: the strain used and mass rearing technique, the radiation dose (and therefore both sterility level and competitiveness of males), the re-mating proportions biased towards wild males. For the latter, we focus on the effect of the release ratio.

Releasing fully sterile males is rarely achieved in SIT programs. Indeed, the dose-response curves for sterility only approach complete sterility asymptotically, and so the elimination of the last 1% residual fertility may require unreasonably high doses [73]. Thus, the appropriate balance between acceptable residual fertility and quality (that is, competitiveness and average lifespan) must be determined. Our simulations show that the necessary release ratio increases dramatically as residual fertility increases. Moreover, when residual fertility is above a certain threshold, determined by 1/N, where N is the basic offspring number, the SIT may not be an efficient control strategy.

It is sometimes recommended that, at the beginning of SIT deployment, competitive males are preferable to completely sterile males, to ensure that most females mate with the released males. Subsequently, fully sterile males become preferable once the wild population density had decreased to a level where less competitive males would significantly outnumber the wild males, compensating for their lower mating success [28]. Use of this model could help operators better plan their SIT implementation, according to the tolerable limit for residual fertility. More work is needed, however, to understand what is the population reduction target that should trigger the change to releases of fully sterile males.

Competitiveness of sterile males against their wild counterparts is important for the success of SIT programs. The use of GRO treatment to enhance the attractiveness of sterile males is now common in SIT programs against medflies. Our simulations clearly show the positive impact of higher competitiveness values on the success of the release campaign. Even with daily releases, the necessary ratios were almost ten times higher for untreated sterile males.

Our numerical outputs confirm that re-mating has contrasting effects on SIT efficiency, but this mainly depends on the biological parameters, the birth-rates and the death-rates, related to the re-mated females compartments, *F*_*WS*_, *F*_*SW*_ and *F*_*WW*_. For the *F*_*WW*_ compartment, re-mating is beneficial since *b*_*WW*_ > *b*_*W*_ and *μ*_*WW*_ < *μ*_*W*_ [46]. One case is always detrimental to SIT (when *ε*_max_ is low and the release ratio large): it is the case where *δ* = 0 (a wild mated female never re-mate) and *δ*_*S*_ > 0 (a female that mated with a sterile male will re-mate): these are the blue curves in all figures. When re-mating is not beneficial for SIT, then the cases no re-mating and equal re-mating provide the same results, while the case with differential re-mating, *δ*_*S*_ > *δ* > 0, provides smaller values *ε*_max_ a bit, and increases the critical release ratio a bit. However, in the case where re-mating has a positive impact on SIT, then equal re-mating and differential re-mating induce an increase of *ε*_max_ and a decrease of the critical release ratio, compared to the other cases, without re-mating, and with sterile female re-mating only. One case, *b*_*WS*_ = *b*_*SW*_ = *b*_*W*_, seems to be more problematic: this is the case where sterile males have no impact on the oviposition rate as they were not able to transfer sterile sperms, such that the double-mated female only use fertile sperms. Our simulations show that, in that case, SIT control is still possible but only with larger release ratios, and very small residual fertility.

Daily versus periodic releases have a considerable impact on the cost of an operational program. Because of the short average lifespan of sterile males considered in the simulations (see Table 2, page 16), a 7-day periodic releases do not appear suitable. Although lifespan is difficult to estimate in the field, one can consider that sterile males are only useful as long as they can inseminate wild females. However, Abraham et al [31] showed that sperm-depleted sterile males would still be able to transfer accessory gland substances that would trigger a refractory behavior in females.

Our model shows the importance of knowing the impact of re-mating because wild and sterile males may not have the same capacity to inseminate females. Costa et al. [74] showed that a (wild) male would use most of its sperm contained in the testes to inseminate one female and a 24h period appears sufficient to replenish the testes with mature sperm [75]. Wild males can inseminate females every day. On the other hand, a sterile male loses the capacity to mature new sperm as the immature cells would have been damaged by the irradiation process; Catala-Oltra et al. [50] showed that 70% of sterile (irradiated at 100 Gy) males mated again 24h after their first mating, and 25% of males mated up to five times. Sterile Queensland flies were reported to have a maximum of 2 or 3 mating capacity [76]; however, even depleted, they were able to copulate and to induce refractoriness in females. Studies have shown that the injection of male accessory gland substances was able to induce refractoriness in Queensland flies, but also in medflies [31, 77]. Costa et al. [74] showed that a lower quantity of sperm transferred would increase the chances of re-mating in females. A more precise understanding of sterile male reproductive capacity could allow optimization of release frequency, ensuring that mating sterile males always saturates the field. While refractoriness studies are useful, our model, and its analysis, shows that it would be equally important to better know about the biological parameters related to double-mated females, like *b*_*WS*_, *b*_*SW*_, *μ*_*WS*_, and *μ*_*SW*_, and also *b*_*WW*_ and *μ*_*WW*_. As showed in (10), we need to estimate these parameters in order to derive the maximal residual fertility, *ε*_max_, and, thus, the critical release ratio.

## 4 Conclusion

The aim of this study was to investigate the impact of re-mating and residual fertility on sterile insect technique efficiency. We assume single- and double-matings. In order to induce a strong Allee effect, that is to stabilize locally asymptotically the origin, $0_{R^{7}}$, we show that the residual fertility parameter, *ε*, has to be lower than a threshold parameter, *ε*_max_, defined in (10), page 9, that depends upon the basic offspring number, N(δ), and also parameters related to double-mated females. From that point of view, our result also encompasses the result found in [20], when no-remating occurs, where we found that *ε* has to fulfil εN(0)<1. Thanks to (10), the larger the basic offspring number, the lower *ε*_max_. However, other parameters are also important in (10): the re-mating rates parameters, *δ* and *δ*_*S*_, and also the birth-rate and the death-rate related to the single- and double-mated fertile females, *F*_*W*_, *F*_*WW*_, *F*_*WS*_, and *F*_*SW*_.

Our theoretical and numerical results also show that re-mating is, in general, not beneficial for SIT, in presence of residual fertility. As the level of residual fertility is related to the radiation dose, our results indicate that a small percentage of residual fertility is not necessarily detrimental to SIT. This is important because the radiation dose has an impact on the lifespan and competitiveness of sterile males. When re-mating is not equal (re-mating is faster after mating with a sterile male), it is necessary to drastically increase the release rate of sterile males.

However, we show, in case 3, that re-mating can be beneficial for SIT by increasing *ε*_max_, in particular when *b*_*WS*_ is substantially smaller than *b*_*W*_. Case 3 is based on a very particular case where the sterile male is from a different genotype. This case could occur in ongoing field studies, where the sterile flies, produced far away from the release place, are not necessary of the same genotype than the local fruit flies. Also, several works [48] have showed that GRO-treatment improves the efficiency of SIT by improving the competitiveness of sterile males [48].

To summarize, our study showed that biological parameters related to re-mating, δ_*_, b_*_, and μ_*_ are central to determine an upper bound for the residual fertility and also the critical release threshold. We found fragmented information in the literature about these parameters. It seems that it would be more than necessary to estimate all these parameters in order to guarantee or enhance the SIT efficacy. We considered here only single and double mating, but our model can be extended to more re-mating, requiring to obtain data related to these events, something which is not easy.

This study was performed within the framework of an SIT feasibility project, CeraTIS-Corse, in Corsica on a 400 ha area of citrus and stone fruits. As mentioned in the introduction, in Corsica, medflies are active from May to December. Since medfly life parameters are known to vary according to the host fruit species, models can consider this variability according to fruit phenology and temperature variation, when available, over the year to obtain a picture of the natural evolution of the population, as in [52] for *Aedes albopictus* in Réunion island. SIT models consider more simplistic population dynamics with averaged parameters over the year; however, it would be interesting to understand whether variation of released male numbers over the year brings a better or similar control while reducing the program costs.

To go further, and in order to optimize the releases ratios, feedback control or mixed-control can be used, like in [20, 26], to adapt the size of the releases along the SIT control, and thus the total cost of the SIT treatment.

Last but not least, it would be interesting to study another SIT control objective, like reducing the wild population below a given economic threshold, like in [52] where mitigating the epidemiological risk, i.e. reducing $R_{0}$, the basic reproduction number, below 1, was possible even if εN>1, in absence of re-mating.

No operational SIT program exists in France yet; however, there is growing interest in making this tool available for farmers as a response to environmental requirements and to the removal of chemical pesticides. The cost-efficiency of SIT will be a crucial element in growers’ adoption of the technique. Therefore, understanding the optimal release strategy that will reduce production costs while ensuring high field efficacy is key. To date, there is no sterile fly production capacity in France, therefore SIT implementation would rely on import from a producing country. It is therefore crucial to determine the limiting factors that may affect release success. This study has shown that field operators should try to gather a better understanding of re-mating occurrence in the field, but should also carefully specify or control the accuracy of the sterilizing dose and consequently the level of residual fertility of the imported sterile flies. Our model being generic, it can be applied to other fruit flies or pests, and, if necessary, with more re-mating.

## Supporting information

S1 Text In Sec. S1, the proof of Theorem 1, page 9, is given. In Sec. S2, we derive the proof of Lemma 2, page 9. In sec. S3, the proof of Proposition 1, page 10, is given. Finally, in Sec. S4, we prove Theorem 2, page 11.
(PDF)
