# Supplementary material for: On the impact of re-mating and residual fertility on the Sterile Insect Technique efficacy: Case study with the medfly, Ceratitis capitata
Source: PLoS Comput Biol. 2024 May 6;20(5):e1012052. doi: 10.1371/journal.pcbi.1012052 (PMC11098522; doi:10.1371/journal.pcbi.1012052)
Supplement: S1 Text — In Sec. S1, the proof of Theorem 1, page 9, is given. In Sec. S2, we derive the proof of Lemma 2, page 9. In sec. S3, the proof of Proposition 1, page 10, is given. Finally, in Sec. S4, we prove Theorem 2, page 11. (PDF) [file pcbi.1012052.s001.pdf]

On the impact of re-mating and residual fertility on the Sterile Insect Technique efficacy: case study with the medfly, *Ceratitis capitata* - Supporting Information

Yves Dumont[1,2,3], Clélia F. Oliva[4¤]

1 UMR AMAP, CIRAD, Saint-Pierre, Réunion island, France
2 UMR AMAP, Univ Montpellier, CIRAD, CNRS, INRAE, IRD, Montpellier, France
3 Department of Mathematics and Applied Mathematics, University of Pretoria, Pretoria, South Africa
4 CTIFL, Bellegarde, France

¤Current Address: TerraTis, Montpellier, France
* yves.dumont@cirad.fr

S1 Proof of Theorem 1

We compute the Jacobian of system (S1)

$$\begin{cases} \dfrac{dA}{dt} = (b_W F_W + b_{WW} F_{WW}) \left(1 - \dfrac{A}{K}\right) - (\nu_A + \mu_A) A \\ \dfrac{dM}{dt} = (1-r)\nu_A A - \mu_M M, \\ \dfrac{dF_W}{dt} = r\nu_A A - (\delta + \mu_F) F_W, \\ \dfrac{dF_{WW}}{dt} = \delta F_W - \mu_{F,WW} F_{WW}, \end{cases} \tag{S1}$$

and we derive

$$J(X) = \begin{pmatrix} -\dfrac{1}{K}(b_W F_W + b_{WW} F_{WW}) - (\nu_A + \mu_A) & 0 & b_W \left(1 - \dfrac{A}{K}\right) & b_{WW}\left(1 - \dfrac{A}{K}\right) \\ (1-r)\nu_A & -\mu_M & 0 & 0 \\ r\nu_A & 0 & -(\mu_F + \delta) & 0 \\ 0 & 0 & \delta & -\mu_{F,WW} \end{pmatrix}$$

Then we set

$$N(X) = \begin{pmatrix} -\dfrac{1}{K}(b_W F_W + b_{WW} F_{WW}) - (\nu_A + \mu_A) & 0 & b_W \left(1 - \dfrac{A}{K}\right) & b_{WW}\left(1 - \dfrac{A}{K}\right) \\ 0 & -\mu_M & 0 & 0 \\ 0 & 0 & -(\mu_F + \delta) & 0 \\ 0 & 0 & 0 & -\mu_{F,WW} \end{pmatrix}$$

and

$$M = \begin{pmatrix} 0 & 0 & 0 & 0 \\ (1-r)\nu_A & 0 & 0 & 0 \\ r\nu_A & 0 & 0 & 0 \\ 0 & 0 & \delta & 0 \end{pmatrix}.$$

Matrix N is Metzler stable and M a nonnegative matrix, such that $M + N$ is a regular splitting [1]. Then

$$-N^{-1} = \begin{pmatrix} \dfrac{1}{(D(F_W,F_{WW})+(\nu_A+\mu_A))} & 0 & \dfrac{b_W\left(1-\dfrac{A}{K}\right)}{(\mu_F+\delta)\,(D(F_W,F_{WW})+(\nu_A+\mu_A))} & \dfrac{b_{WW}\left(1-\dfrac{A}{K}\right)}{\mu_{F,WW}\,(D(F_W,F_{WW})+(\nu_A+\mu_A))} \\ 0 & \dfrac{1}{\mu_M} & 0 & \\ & 0 & \dfrac{1}{(\mu_F+\delta)} & 0 \\ 0 & 0 & 0 & \dfrac{1}{\mu_{F,WW}} \end{pmatrix},$$

with $D(F_W,F_{WW}) = \dfrac{1}{K}\left(b_W F_W + b_{WW}F_{WW}\right)$. Thus

$$-N^{-1}(X)M = \begin{pmatrix} \dfrac{r\nu_A b_W\left(1-\dfrac{A}{K}\right)}{(\mu_F+\delta)\,(D(F_W,F_{WW})+(\nu_A+\mu_A))} & 0 & \dfrac{\delta b_{WW}\left(1-\dfrac{A}{K}\right)}{\mu_{F,WW}\,(D(F_W,F_{WW})+(\nu_A+\mu_A))} & 0 \\ \dfrac{(1-r)\,\nu_A}{\mu_M} & 0 & 0 & 0 \\ \dfrac{r\nu_A}{\mu_F+\delta} & 0 & 0 & 0 \\ 0 & 0 & \dfrac{\delta}{\mu_{F,WW}} & 0 \end{pmatrix}.$$

Thus, the characteristic polynomial becomes

$$p(\lambda) = \lambda^2\left(\lambda^2 - \lambda\frac{r\nu_A b_W\left(1-\dfrac{A}{K}\right)}{(\mu_F+\delta)\,(D(F_W,F_{WW})+(\nu_A+\mu_A))} - \right.$$
$$\left. - \frac{r\nu_A}{\mu_F+\delta}\frac{\delta b_{WW}\left(1-\dfrac{A}{K}\right)}{\mu_{F,WW}\,(D(F_W,F_{WW})+(\nu_A+\mu_A))}\right).$$

We recall that, for a second order polynomial $q(z) = z^2 + a_1 z + a_2$, to show that all roots are in the inside the unit disc, the Jury criterion leads to the following necessary conditions

$$\begin{cases} q(1) > 0, \\ q(-1) > 0, \end{cases} \tag{S2}$$

and a sufficient condition

$$|a_2| < 1. \tag{S3}$$

- When $E = \mathbf{0}$, then

$$q(\lambda) = \lambda^2 - \lambda\frac{r\nu_A b_W}{(\mu_F+\delta)\,(\nu_A+\mu_A)} - \frac{r\nu_A}{\mu_F+\delta}\frac{\delta b_{WW}}{\mu_{F,WW}\,(\nu_A+\mu_A)},$$

  such that

$$q(1) = 1 - \frac{r\nu_A b_W}{(\mu_F+\delta)\,(\nu_A+\mu_A)} - \frac{r\nu_A}{\mu_F+\delta}\frac{\delta b_{WW}}{\mu_{F,WW}\,(\nu_A+\mu_A)} = 1 - \mathcal{R}(\delta) > 0,$$

  when $\mathcal{R}(\delta) < 1$. From $q(1) > 0$, we deduce that

$$a_0 = \frac{r\nu_A}{\mu_F+\delta}\frac{\delta b_{WW}\left(1-\dfrac{A}{K}\right)}{\mu_{F,WW}\left(\dfrac{1}{K}\left(b_W F_W + b_{WW}F_{WW}\right)+(\nu_A+\mu_A)\right)} < 1.$$

In addition

$$q(-1) = 1 + \frac{r\nu_A b_W}{(\mu_F + \delta)(\nu_A + \mu_A)} - \frac{r\nu_A}{\mu_F + \delta}\frac{\delta b_{WW}}{\mu_{F,WW}(\nu_A + \mu_A)} > 0.$$

Thus, we deduce that $\rho(-N^{-1}(\mathbf{0})M) < 1$, which implies that $s(J(\mathbf{0}) < 0$. Thus the trivial equilibrium is $\mathbf{0}$ is LAS iff $\mathcal{R}(\delta) < 1$ for system (S1).

- Assuming $\mathcal{R} > 1$, then, it is straightforward to show that an endemic equilibrium $\mathbf{E}$ exists with

$$\begin{cases} A_0^* = \left(1 - \frac{1}{\mathcal{R}}\right) K, \\ M_0^* = \frac{(1-r)\nu_A}{\mu_M}\left(1 - \frac{1}{\mathcal{R}}\right) K, \\ F_{0,W}^* = \dfrac{r\nu_A}{\delta + \mu_F}\left(1 - \frac{1}{\mathcal{R}}\right) K, \\ F_{0,WW}^* = \dfrac{\delta}{\mu_{F,WW}}\dfrac{r\nu_A}{\delta + \mu_F}\left(1 - \frac{1}{\mathcal{R}}\right) K, \end{cases}$$

such that the characteristic equation becomes

$$p[\lambda] = \lambda^2 q_{E^*}(\lambda),$$

with

$$q_{E^*}(\lambda) = \lambda^2 - \frac{\mathcal{N}}{\mathcal{R}(\mathcal{R}+1)}\lambda - \frac{\delta b_{WW}}{\mu_{F,WW} b_W}\frac{\mathcal{N}}{\mathcal{R}(\mathcal{R}+1)}.$$

We have

$$q_E(1) = 1 - \frac{\mathcal{N}}{\mathcal{R}(\mathcal{R}+1)}\left(1 + \frac{\delta b_{WW}}{\mu_{F,WW} b_W}\right) = 1 - \frac{1}{\mathcal{R}+1} > 0,$$

because $\mathcal{R} > 0$, which implies that

$$\frac{\delta b_{WW}}{\mu_{F,WW} b_W}\frac{\mathcal{N}}{\mathcal{R}(\mathcal{R}+1)} < 1,$$

and also

$$q_E(-1) = 1 + \frac{\mathcal{N}}{\mathcal{R}(\mathcal{R}+1)} - \frac{\delta b_{WW}}{\mu_{F,WW} b_W}\frac{\mathcal{N}}{\mathcal{R}(\mathcal{R}+1)} > 0.$$

Thus, we deduce that $\rho(-N^{-1}(\mathbf{E})M) < 1$, which implies that $s(J(\mathbf{E}) < 0$. Thus the trivial equilibrium is $\mathbf{E}$ is LAS iff $\mathcal{R}(\delta) > 1$ for system (S1).

From the Theory of Monotone Cooperative system [2], and following [3, Theorem 6] or [4, Theorem 1], we know that once an equilibrium exists, is unique and LAS, then it is GAS. Thus, our results follow.

S2 Proof of Lemma 2

We compute the Jacobian related to system (2) at $\mathbf{0}_{\mathbb{R}^7}$

$$J(\mathbf{0}_{\mathbb{R}^7}) = \begin{pmatrix} -(\nu_A + \mu_A) & 0 & b_W & 0 & b_{WS} & b_{SW} & b_{WW} \\ (1-r)\nu_A & -\mu_M & 0 & 0 & 0 & 0 & 0 \\ \varepsilon r\nu_A & 0 & -(\delta + \mu_F) & 0 & 0 & 0 & 0 \\ (1-\varepsilon) r\nu_A & 0 & 0 & -(\delta_S + \mu_F) & 0 & 0 & 0 \\ 0 & 0 & (1-\varepsilon)\delta & 0 & -\mu_{F,WS} & 0 & 0 \\ 0 & 0 & 0 & \varepsilon\delta_S & & -\mu_{F,SW} & 0 \\ 0 & 0 & \varepsilon\delta & 0 & 0 & 0 & -\mu_{F,WW} \end{pmatrix} \quad \text{(S4)}$$

Setting

$$M = \begin{pmatrix} 0 & 0 & 0 & 0 & 0 & 0 & 0 \\ (1-r)\,\nu_A & 0 & 0 & 0 & 0 & 0 & 0 \\ \varepsilon r \nu_A & 0 & 0 & 0 & 0 & 0 & 0 \\ (1-\varepsilon)\, r\nu_A & 0 & 0 & 0 & 0 & 0 & 0 \\ 0 & 0 & (1-\varepsilon)\,\delta & 0 & 0 & 0 & 0 \\ 0 & 0 & 0 & \varepsilon\delta_S & 0 & 0 & 0 \\ 0 & 0 & \varepsilon\delta & 0 & 0 & 0 & 000 \end{pmatrix}$$

and

$$N = \begin{pmatrix} -(\nu_A+\mu_A) & 0 & b_W & 0 & b_{WS} & b_{SW} & b_{WW} \\ 0 & -\mu_M & 0 & 0 & 0 & 0 & 0 \\ 0 & 0 & -(\delta+\mu_F) & 0 & 0 & 0 & 0 \\ 0 & 0 & 0 & -(\delta_S+\mu_F) & 0 & 0 & 0 \\ 0 & 0 & 0 & 0 & -\mu_{F,WS} & 0 & 0 \\ 0 & 0 & 0 & 0 & 0 & -\mu_{F,SW} & 0 \\ 0 & 0 & 0 & 0 & 0 & 0 & -\mu_{F,WW} \end{pmatrix},$$

where N is an invertible M-matrix and M a nonnegative matrix, then, we deduce

$$-N^{-1}M = \begin{pmatrix} \frac{b_W \varepsilon r\nu_A}{(\nu_A+\mu_A)(\delta+\mu_F)} & 0 & \frac{b_{W,S}(1-\varepsilon)\delta}{(\nu_A+\mu_A)\mu_{F,WS}} + \frac{b_{WW}\varepsilon\delta}{(\nu_A+\mu_A)\mu_{F,WW}} & \frac{b_{S,W}\varepsilon\delta_S}{(\nu_A+\mu_A)\mu_{F,SW}} & 0 & 0 & 0 \\ \frac{(1-r)\nu_A}{\mu_M} & 0 & 0 & 0 & 0 & 0 & 0 \\ \frac{\varepsilon r\nu_A}{(\delta+\mu_F)} & 0 & 0 & 0 & 0 & 0 & 0 \\ \frac{(1-\varepsilon) r\nu_A}{(\delta_S+\mu_F)} & 0 & 0 & 0 & 0 & 0 & 0 \\ 0 & 0 & \frac{(1-\varepsilon)\delta}{\mu_{F,WS}} & 0 & 0 & 0 & 0 \\ 0 & 0 & 0 & \frac{\varepsilon\delta_S}{\mu_{F,SW}} & 0 & 0 & 0 \\ 0 & 0 & \frac{\varepsilon\delta}{\mu_{F,WW}} & 0 & 0 & 0 & 0 \end{pmatrix}$$

After some computations, we can show that the characteristic polynomial of $-N^{-1}M$ reduces to $-\lambda^5 q(\lambda)$, where

$$q(\lambda) = \lambda^2 - \lambda\varepsilon\mathcal{N}(\delta) - \varepsilon\mathcal{N}(\delta)\left(\varepsilon\frac{\delta}{\mu_{F,WW}}\frac{b_{W,W}}{b_W} + (1-\varepsilon)\left(\frac{\delta_S}{\mu_{F,SW}}\frac{b_{S,W}}{b_W}\frac{\delta+\mu_F}{\delta_S+\mu_F} + \frac{\delta}{\mu_{F,WS}}\frac{b_{W,S}}{b_W}\right)\right).$$

To show that all roots, λ^*, are such that $|\lambda^*| < 1$, we have to check Jury's criterion (see (S2) and (S3)), namely $q(1) > 0$, $q(-1) > 0$ and

$$\varepsilon\mathcal{N}(\delta)\left(\varepsilon\frac{\delta}{\mu_{F,WW}}\frac{b_{W,W}}{b_W} + (1-\varepsilon)\left(\frac{\delta_S}{\mu_{F,SW}}\frac{b_{S,W}}{b_W}\frac{\delta+\mu_F}{\delta_S+\mu_F} + \frac{\delta}{\mu_{F,WS}}\frac{b_{W,S}}{b_W}\right)\right) < 1.$$

It is easy to check that $q(-1) > 0$. Then

$$q(1) = 1 - \varepsilon\mathcal{N}(\delta)\left(1 + \varepsilon\frac{\delta}{\mu_{F,WW}}\frac{b_{W,W}}{b_W} + (1-\varepsilon)\left(\frac{\delta_S}{\mu_{F,SW}}\frac{b_{S,W}}{b_W}\frac{\delta+\mu_F}{\delta_S+\mu_F} + \frac{\delta}{\mu_{F,WS}}\frac{b_{W,S}}{b_W}\right)\right) > 0, \tag{S5}$$

if and only if

$$\varepsilon\mathcal{N}(\delta)\left(1 + \varepsilon\frac{\delta}{\mu_{F,WW}}\frac{b_{W,W}}{b_W} + (1-\varepsilon)\left(\frac{\delta_S}{\mu_{F,SW}}\frac{b_{S,W}}{b_W}\frac{\delta+\mu_F}{\delta_S+\mu_F} + \frac{\delta}{\mu_{F,WS}}\frac{b_{W,S}}{b_W}\right)\right) - \frac{1}{\mathcal{N}} < 0. \tag{S6}$$

Thus we have to find under what condition the previous polynomial in ε takes negative values. After straightforward calculations, we found that, for $0 \leq \varepsilon \leq \varepsilon_{\max}$, with $\varepsilon_{\max} =$

$$\frac{2}{\mathcal{N}(\delta)} \frac{1}{\left(1+\frac{\delta_S}{\mu_{F,SW}}\frac{b_{S,W}}{b_W}\frac{\delta+\mu_F}{\delta_S+\mu_F}+\frac{\delta}{\mu_{F,WS}}\frac{b_{W,S}}{b_W}\right)\left(\sqrt{1+\frac{4}{\mathcal{N}(\delta)}\frac{\left(\frac{\delta}{b_W}\left(\frac{b_{W,W}}{\mu_{F,WW}}-\frac{b_{W,S}}{\mu_{F,WS}}\right)-\frac{\delta_S}{\mu_{F,WS}}\frac{b_{S,W}}{b_W}\frac{\delta+\mu_F}{\delta_S+\mu_F}\right)}{\left(1+\frac{\delta_S}{\mu_{F,SW}}\frac{b_{S,W}}{b_W}\frac{\delta+\mu_F}{\delta_S+\mu_F}+\frac{\delta}{\mu_{F,WS}}\frac{b_{W,S}}{b_W}\right)^2}}+1\right)}, \quad \text{(S7)}$$

condition (S6) is verified. Thus we conclude that $\rho(-N^{-1}M) < 1$ which implies $s(J(\mathbf{0}_{\mathbb{R}^7})) < 0$, and the result follows.

S3 Proof of Proposition 1

We are looking for a condition to have at least one positive equilibrium.Thus, we have to solve

$$\begin{cases} (b_W F_W + b_{W,W} F_{W,W} + b_{WS} F_{W,S} + b_{S,W} F_{S,W}) \left(1 - \frac{A}{K}\right) = (\nu_A + \mu_A) A \\ (1-r)\,\nu_A A = \mu_M M, \\ r\nu_A \frac{M + \varepsilon\gamma M_S}{M + \gamma M_S} A = (\delta + \mu_F) F_W, \\ r\nu_A \frac{\gamma M_S (1-\varepsilon)}{M + \gamma M_S} A = (\delta_S + \mu_F) F_S, \\ \frac{\gamma M_S (1-\varepsilon)}{M + \gamma M_S} \delta F_W = \mu_{F,WS} F_{W,S} \\ \frac{M + \varepsilon\gamma M_S}{M + \gamma M_S} \delta_S F_S = \mu_{F,WS} F_{S,W} \\ \frac{M + \varepsilon\gamma M_S}{M + \gamma M_S} \delta F_W = \mu_{F,WW} F_{W,W} \end{cases}$$

such that

$$F_W = \frac{r\nu_A}{\delta + \mu_F} \frac{M + \varepsilon\gamma M_S}{M + \gamma M_S} A,$$

$$F_S = \frac{r\nu_A}{\delta_S + \mu_F} \frac{(1+\varepsilon)\gamma M_S}{M + \gamma M_S} A,$$

$$F_{W,W} = \frac{\delta}{\mu_{F,WW}} \frac{M + \varepsilon\gamma M_S}{M + \gamma M_S} F_W = \frac{\delta}{\mu_{F,WW}} \frac{r\nu_A}{\delta + \mu_F} \left(\frac{M + \varepsilon\gamma M_S}{M + \gamma M_S}\right)^2 A,$$

$$F_{W,S} = \frac{M + \varepsilon\gamma M_S}{M + \gamma M_S} \frac{(1-\varepsilon)\gamma M_S}{M + \gamma M_S} \frac{\delta}{\mu_{F,WS}} \frac{r\nu_A}{\delta + \mu_F} A$$

$$F_{S,W} = \frac{M + \varepsilon\gamma M_S}{M + \gamma M_S} \frac{(1-\varepsilon)\gamma M_S}{M + \gamma M_S} \frac{\delta_S}{\mu_{F,SW}} \frac{r\nu_A}{\delta_S + \mu_F} A.$$

Thus from the first equation

$$b_W F_W + b_{W,W} F_{W,W} + b_{WS} F_{W,S} + b_{S,W} F_{S,W} =$$

$$= \left[\frac{b_W r\nu_A}{\delta + \mu_F} \frac{M + \varepsilon\gamma M_S}{M + \gamma M_S} + \frac{\delta}{\mu_{F,WW}} \frac{b_{W,W} r\nu_A}{\delta + \mu_F} \left(\frac{M + \varepsilon\gamma M_S}{M + \gamma M_S}\right)^2 + \right.$$
$$\left. + \frac{M + \varepsilon\gamma M_S}{M + \gamma M_S} \frac{(1-\varepsilon)\gamma M_S}{M + \gamma M_S} \left(\frac{\delta}{\mu_{F,WS}} \frac{b_{W,S} r\nu_A}{\delta + \mu_F} + \frac{\delta_S}{\mu_{F,SW}} \frac{b_{S,W} r\nu_A}{\delta_S + \mu_F}\right)\right] A$$

that is

$$\frac{1}{(\nu_A+\mu_A)}\left[\frac{b_W r\nu_A}{\delta+\mu_F}\left(M+\varepsilon\gamma M_S\right)\left(M+\gamma M_S\right)+\frac{\delta}{\mu_{F,WW}}\frac{b_{WW} r\nu_A}{\delta+\mu_F}\left(M+\varepsilon\gamma M_S\right)^2+\right.$$
$$\left.+(1-\varepsilon)\gamma M_S\left(M+\varepsilon\gamma M_S\right)\left(\frac{\delta_S}{\mu_{F,SW}}\frac{b_{SW} r\nu_A}{\delta_S+\mu_F}+\frac{\delta}{\mu_{F,WS}}\frac{b_{WS} r\nu_A}{\delta+\mu_F}\right)\right]\times$$
$$\times\left(1-\frac{\mu_M}{(1-r)\nu_A K}M\right)=\left(M+\gamma M_S\right)^2,$$

that is

$$\left[\left(M+\varepsilon\gamma M_S\right)\left(M+\gamma M_S\right)+\frac{\delta}{\mu_{F,WW}}\frac{b_{WW}}{b_W}\left(M+\varepsilon\gamma M_S\right)^2+\right.$$
$$\left.+(1-\varepsilon)\gamma M_S\left(M+\varepsilon\gamma M_S\right)\left(\frac{\delta_S}{\mu_{F,SW}}\frac{\delta+\mu_F}{\delta_S+\mu_F}\frac{b_{SW}}{b_W}+\frac{\delta}{\mu_{F,WS}}\frac{b_{WS}}{b_W}\right)\right]\times$$
$$\times\left(1-\frac{\mu_M}{(1-r)\nu_A K}M\right)=\frac{1}{\mathcal{N}}\left(M+\gamma M_S\right)^2.$$

Expanding the left-hand side leads to

$$M^2\left(1+\frac{\delta}{\mu_{F,WW}}\frac{b_{W,W}}{b_W}\right)+M\gamma M_S\left(1+\varepsilon\left(1+2\frac{\delta}{\mu_{F,WW}}\frac{b_{W,W}}{b_W}\right)+\right.$$
$$\left.+(1-\varepsilon)\left(\frac{\delta_S}{\mu_{F,SW}}\frac{b_{SW}}{b_W}\frac{\delta+\mu_F}{\delta_S+\mu_F}+\frac{\delta_S}{\mu_{F,WS}}\frac{b_{WS}}{b_W}\right)\right)+$$
$$+\varepsilon\left(1+\varepsilon\frac{\delta}{\mu_{F,WW}}\frac{b_{W,W}}{b_W}+(1-\varepsilon)\left(\frac{\delta_S}{\mu_{F,SW}}\frac{b_{SW}}{b_W}\frac{\delta+\mu_F}{\delta_S+\mu_F}+\frac{\delta_S}{\mu_{F,WS}}\frac{b_{WS}}{b_W}\right)\right)\left(\gamma M_S\right)^2$$

We have

$$Q(M)\left(1-\frac{\mu_M}{(1-r)\nu_A K}M\right)=\frac{1}{\mathcal{N}(\delta)}(M+\gamma M_S)^2,$$

with

$$Q(M)=M^2\left(1+\frac{\delta}{\mu_{F,WW}}\frac{b_{W,W}}{b_W}\right)+\left(1+\varepsilon\left(1+2\frac{\delta}{\mu_{F,WW}}\frac{b_{W,W}}{b_W}\right)+\right.$$
$$\left.+(1-\varepsilon)\left(\frac{\delta_S}{\mu_{F,SW}}\frac{b_{SW}}{b_W}\frac{\delta+\mu_F}{\delta_S+\mu_F}+\frac{\delta_S}{\mu_{F,WS}}\frac{b_{WS}}{b_W}\right)\right)\gamma M_S M+$$
$$+\varepsilon\left(1+\varepsilon\frac{\delta}{\mu_{F,WW}}\frac{b_{W,W}}{b_W}+(1-\varepsilon)\left(\frac{\delta_S}{\mu_{F,SW}}\frac{b_{SW}}{b_W}\frac{\delta+\mu_F}{\delta_S+\mu_F}+\frac{\delta_S}{\mu_{F,WS}}\frac{b_{WS}}{b_W}\right)\right)\left(\gamma M_S\right)^2.$$

The roots of Q are

$$M_1^*=-\frac{\gamma M_S}{1+\frac{\delta}{\mu_{F,WW}}\frac{b_{W,W}}{b_W}}\left(1+\varepsilon\frac{\delta}{\mu_{F,WW}}\frac{b_{W,W}}{b_W}+(1-\varepsilon)\left(\frac{\delta_S}{\mu_{F,SW}}\frac{b_{SW}}{b_W}\frac{\delta+\mu_F}{\delta_S+\mu_F}+\frac{\delta_S}{\mu_{F,WS}}\frac{b_{WS}}{b_W}\right)\right. \tag{S8}$$

and $M_2^*=-\varepsilon\gamma M_S<0$. They are both real negative. Thus we are looking for positive roots of

$$\Psi(M)\equiv\left(M-M_1^*\right)\left(M-M_2^*\right)\left(1-\frac{\mu_M}{(1-r)\nu_A K}M\right)=\frac{1}{\mathcal{R}(\delta)}(M+\gamma M_S)^2\equiv\Phi(M). \tag{S9}$$

Thus, we can derive 3 cases as showed in Fig 1, page 7:

1. Since $\Phi(0) < \Psi(0)$, then there always exists a positive root, M^*. Note also that $\Phi(0) \leq \Psi(0)$ is equivalent to choose ε such that

$$\varepsilon\left(1+\varepsilon\frac{\delta}{\mu_{F,WW}}\frac{b_{W,W}}{b_W}+(1-\varepsilon)\left(\frac{\delta_S}{\mu_{F,SW}}\frac{b_{S,W}}{b_W}\frac{\delta+\mu_F}{\delta_S+\mu_F}+\frac{\delta}{\mu_{F,WS}}\frac{b_{W,S}}{b_W}\right)\right)>\frac{1}{\mathcal{N}}$$

which is the opposite condition of (S6), page 4, that is $\varepsilon > \varepsilon_{\max}$, where $\varepsilon_{\max}$ given in (S7), page 5, and for which equilibrium $\mathbf{0}_{\mathbb{R}^7}$ is unstable.

2. Since $\Phi(0) \geq \Psi(0)$, that is $\varepsilon \in [0, \varepsilon_{\max}]$, we have two cases

   (a) either γM_S is small such that we can have one intersection or two intersections between Ψ and Φ

   (b) either γM_S is sufficiently large such that Ψ and Φ do not intersect.

   Thus, we can deduce that there exists a critical threshold $\Lambda_{\varepsilon,cont}^{crit} = \mu_S M_S^{crit}$ such that above this critical threshold no positive equilibrium can exists, only the trivial equilibrium, i.e. $\mathbf{0}_{\mathbb{R}^7}$. We are not able to derive a formula for M_S^{crit}, and thus for $\Lambda_{\varepsilon,cont}^{crit}$, but we can solve (S9), page 6, to find it.

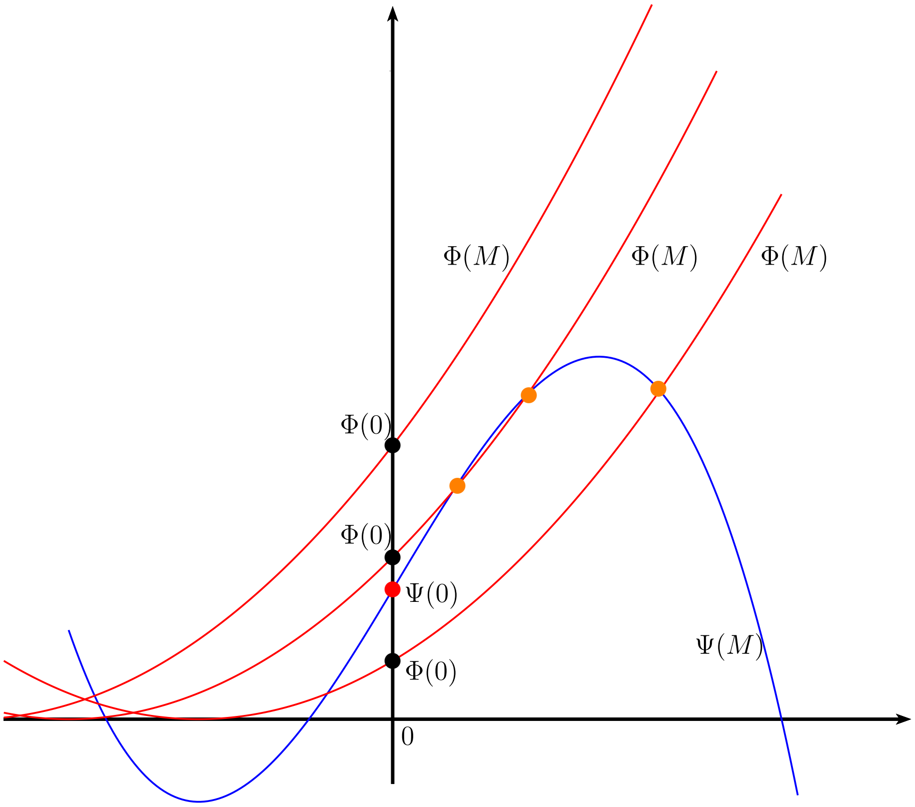

Fig 1. Intersections between Ψ (in blue) and Φ (in red) - 3 cases with no, one or two intersections (orange bullets).

In the case where $\varepsilon = \varepsilon_{\max}$, we can derive an explicit formula for $\Lambda_{\varepsilon,cont}^{crit}$. Indeed, we have $M_1^* M_2^* = m_1^*(\varepsilon_{\max}) * \varepsilon_{\max}(\gamma M_S)^2 = \dfrac{1}{\mathcal{R}}(\gamma M_S)^2$, that is $m_1^*(\varepsilon_{\max})\varepsilon_{\max} = \dfrac{1}{\mathcal{R}}$ for all

$M_S > 0$, with $m_1^*(\varepsilon) =$

$$\frac{1}{1+\frac{\delta}{\mu_{F,WW}}\frac{b_{W,W}}{b_W}}\left(1+\varepsilon\frac{\delta}{\mu_{F,WW}}\frac{b_{W,W}}{b_W}+(1-\varepsilon)\left(\frac{\delta_S}{\mu_{F,SW}}\frac{b_{SW}}{b_W}\frac{\delta+\mu_F}{\delta_S+\mu_F}+\frac{\delta_S}{\mu_{F,WS}}\frac{b_{WS}}{b_W}\right)\right).$$

Then, equation (S9) becomes

$$(M+\frac{1}{\varepsilon_{\max}\mathcal{R}(\delta)}\gamma M_S)\,(M+\varepsilon_{\max}\gamma M_S)\left(1-\frac{\mu_M}{(1-r)\nu_A K}M\right)=\frac{1}{\mathcal{R}(\delta)}(M+\gamma M_S)^2,$$

and it reduces to the following, equivalent, second order equation to solve

$$\begin{aligned}&-\frac{\mu_M}{(1-r)\nu_A K}M^2+\left(\left(1-\frac{1}{\mathcal{R}(\delta)}\right)-\frac{\mu_M}{(1-r)\nu_A K}\left(\frac{1}{\varepsilon_{\max}\mathcal{R}(\delta)}+\varepsilon_{\max}\right)\gamma M_S\right)M+\\&+\left(\frac{1}{\varepsilon_{\max}\mathcal{R}(\delta)}+\varepsilon_{\max}-\frac{2}{\mathcal{R}(\delta)}-\frac{1}{\mathcal{R}(\delta)}\frac{\mu_M}{(1-r)\nu_A K}\gamma M_S\right)\gamma M_S=0.\end{aligned}$$

Computing the discriminant leads to

$$\Delta=\left(\frac{\mu_M}{(1-r)\nu_A K}\right)^2\left(\frac{1}{\varepsilon_{\max}\mathcal{R}(\delta)}-\varepsilon_{\max}\right)^2(\gamma M_S)^2-$$

$$-2\left(1-\frac{1}{\mathcal{R}(\delta)}\right)\frac{\mu_M}{(1-r)\nu_A K}\left(\frac{1}{\varepsilon_{\max}\mathcal{R}(\delta)}+\varepsilon_{\max}\right)\gamma M_S+\left(1-\frac{1}{\mathcal{R}(\delta)}\right)^2,$$

that is a second-order polynomial in γM_S, for which the discriminant is

$$\delta=16\left(\frac{\mu_M}{(1-r)\nu_A K}\right)^2\left(1-\frac{1}{\mathcal{R}(\delta)}\right)^2\frac{1}{\mathcal{R}(\delta)}.$$

Thus we derive the first positive root

$$\gamma M^*_{T,crit}=\frac{\left(1-\frac{1}{\mathcal{R}(\delta)}\right)}{\frac{\mu_M}{(1-r)\nu_A K}\left(\frac{1}{\varepsilon_{\max}\mathcal{R}(\delta)}+\varepsilon_{\max}+2\sqrt{\frac{1}{\mathcal{R}(\delta)}}\right)},$$

from which we deduce

$$\gamma\Lambda^{crit}_{\varepsilon,cont}=\frac{\mu_S}{\frac{1}{\varepsilon_{\max}\mathcal{R}(\delta)}+\varepsilon_{\max}+2\sqrt{\frac{1}{\mathcal{R}(\delta)}}}M_0^*,$$

related to the initial male population at equilibrium, M_0^*.

S4 Proof of Theorem 2

We have the following system

$$\begin{cases}
\dfrac{dA}{dt} = \left(b_W F_W + b_{WW} F_{WW} + b_{SW} F_{SW} + b_{WS} F_{WS}\right)\left(1 - \dfrac{A}{K}\right) - \left(\nu_A + \mu_A\right) A, \\
\dfrac{dM}{dt} = \left(1 - r\right)\nu_A A - \mu_M M, \\
\dfrac{dF_W}{dt} = r\nu_A \dfrac{M + \varepsilon\gamma M_S^*}{M + \gamma M_S^*} A - \left(\delta + \mu_F\right) F_W, \\
\dfrac{dF_S}{dt} = r\nu_A \dfrac{(1-\varepsilon)\gamma M_S^*}{M + \gamma M_S^*} A - \left(\delta_S + \mu_F\right) F_S, \\
\dfrac{dF_{WW}}{dt} = \delta \dfrac{M + \varepsilon\gamma M_S^*}{M + \gamma M_S^*} F_W - \mu_{F,WW} F_{WW}, \\
\dfrac{dF_{WS}}{dt} = \delta \dfrac{(1-\varepsilon)\gamma M_S^*}{M + \gamma M_S^*} F_W - \mu_{F,WS} F_{WS}, \\
\dfrac{dF_{SW}}{dt} = \delta_S \dfrac{M + \varepsilon\gamma M_S^*}{M + \gamma M_S^*} F_S - \mu_{F,SW} F_{SW}.
\end{cases} \tag{S10}$$

Even if system (S10) is not cooperative, it can be studied using the monotone theory approach [2]. Indeed, since $\dfrac{\gamma M_S^*}{M + \gamma M_S^*} \leq 1$ in equations $(S10)_{4,6}$, it is straightforward to show that system (S10) is upper bounded by the following monotone cooperative auxiliary system [2]:

$$\begin{cases}
\dfrac{dA}{dt} = \left(b_W F_W + b_{WW} F_{WW} + b_{SW} F_{SW} + b_{WS} F_{WS}\right)\left(1 - \dfrac{A}{K}\right) - \left(\nu_A + \mu_A\right) A, \\
\dfrac{dM}{dt} = \left(1 - r\right)\nu_A A - \mu_M M, \\
\dfrac{dF_W}{dt} = r\nu_A \dfrac{M + \varepsilon\gamma M_S^*}{M + \gamma M_S^*} A - \left(\delta + \mu_F\right) F_W, \\
\dfrac{dF_S}{dt} = r\nu_A (1-\varepsilon) A - \left(\delta_S + \mu_F\right) F_S, \\
\dfrac{dF_{WW}}{dt} = \delta \dfrac{M + \varepsilon\gamma M_S^*}{M + \gamma M_S^*} F_W - \mu_{F,WW} F_{WW}, \\
\dfrac{dF_{WS}}{dt} = \delta (1-\varepsilon) F_W - \mu_{F,WS} F_{WS}, \\
\dfrac{dF_{SW}}{dt} = \delta_S \dfrac{M + \varepsilon\gamma M_S}{M + \gamma M_S} F_S - \mu_{F,SW} F_{SW}.
\end{cases} \tag{S11}$$

We first check the condition on ε to have $\mathbf{0}_{\mathbb{R}^7}$ LAS for the monotone cooperative system (S11), page 9. We compute

$$J(\mathbf{0}_{\mathbb{R}^7}) = \begin{pmatrix}
-\left(\nu_A + \mu_A\right) & 0 & b_W & 0 & b_{WS} & b_{SW} & b_{WW} \\
\left(1 - r\right)\nu_A & -\mu_M & 0 & 0 & 0 & 0 & 0 \\
\varepsilon r\nu_A & 0 & -\left(\delta + \mu_F\right) & 0 & 0 & 0 & 0 \\
\left(1-\varepsilon\right) r\nu_A & 0 & 0 & -\left(\delta_S + \mu_F\right) & 0 & 0 & 0 \\
0 & 0 & \left(1-\varepsilon\right)\delta & 0 & -\mu_{F,WS} & 0 & 0 \\
0 & 0 & 0 & \varepsilon\delta_S & & -\mu_{F,SW} & 0 \\
0 & 0 & \varepsilon\delta & 0 & 0 & 0 & -\mu_{F,WW}
\end{pmatrix} \tag{S12}$$

We observe that system (S10) and system (S11) have the same Jacobian at $\mathbf{0}_{\mathbb{R}^7}$, such that, following section S2, we can deduce that $\mathbf{0}_{\mathbb{R}^7}$ is also LAS for system (S11) when $\varepsilon \leq \varepsilon_{\max}$, and unstable otherwise.

Following the same reasoning than in section S3, we show that a positive equilibrium

exists for model (S11) if there exists positive roots of

$$\Psi_U(M) \equiv \left(M - M_{1,U}^*\right)\left(M - M_2^*\right)\left(1 - \frac{\mu_M}{(1-r)\nu_A K} M\right) = \frac{1}{\mathcal{R}_\varepsilon(\delta)}(M+\gamma M_S)^2 \equiv \Phi(M), \tag{S13}$$

where $M_2^* = -\varepsilon\gamma M_S < 0$ and

$$M_{1,U}^* = -\frac{\left(1 + \varepsilon\frac{\delta}{\mu_{F,WW}}\frac{b_{W,W}}{b_W} + (1-\varepsilon)\left(\frac{\delta_S}{\mu_{F,SW}}\frac{b_{SW}}{b_W}\frac{\delta+\mu_F}{\delta_S+\mu_F} + \frac{\delta_S}{\mu_{F,WS}}\frac{b_{WS}}{b_W}\right)\right)}{1 + \frac{b_{WW}}{b_W}\frac{\delta}{\mu_{F,WW}} + (1-\varepsilon)\left(\frac{b_{SW}}{b_W}\frac{\delta_S}{\mu_{F,SW}}\frac{\delta+\mu_F}{(\delta_S+\mu_F)} + \frac{b_{WS}}{b_W}\frac{\delta}{\mu_{F,WS}}\right)}\gamma M_S < 0,$$

and

$$\mathcal{R}_\varepsilon(\delta) = \mathcal{N}(\delta)\left(1 + \frac{b_{WW}}{b_W}\frac{\delta}{\mu_{F,WW}} + (1-\varepsilon)\left(\frac{b_{SW}}{b_W}\frac{\delta_S}{\mu_{F,SW}}\frac{\delta+\mu_F}{(\delta_S+\mu_F)} + \frac{b_{WS}}{b_W}\frac{\delta}{\mu_{F,WS}}\right)\right)$$

Notice that $M_{1,U}^* > M_1^*$, where M_1^* is defined in (S8). Like in section S3, we derive 3 cases, as showed in Fig. 1, page 7, where Ψ is replaced by Ψ_U.

1. Since $\Phi_0(0) < \Psi(0)$, then (S13) always admits a positive root, M^*. Note also that $\Phi_U(0) \leq \Psi(0)$ is equivalent to choose ε such that

$$\varepsilon\left(1 + \varepsilon\frac{\delta}{\mu_{F,WW}}\frac{b_{W,W}}{b_W} + (1-\varepsilon)\left(\frac{\delta_S}{\mu_{F,SW}}\frac{b_{S,W}}{b_W}\frac{\delta+\mu_F}{\delta_S+\mu_F} + \frac{\delta}{\mu_{F,WS}}\frac{b_{W,S}}{b_W}\right)\right) > \frac{1}{\mathcal{N}}$$

   which is the opposite condition of (S6), page 4, that is $\varepsilon > \varepsilon_{\max}$, where $\varepsilon_{\max}$ is given in (S7), page 5, and for which equilibrium $\mathbf{0}_{\mathbb{R}^7}$ is unstable for system (S11).

2. Since $\Phi_U(0) \geq \Psi(0)$, that is $\varepsilon \in [0, \varepsilon_{\max}]$, we have two cases

   (a) either γM_S is small such that we can have one intersection or two intersections between Ψ_U and Φ

   (b) either γM_S is sufficiently large such that Ψ_U and Φ do not intersect.

   Thus, we can deduce that there exists a critical threshold $\Lambda_{\varepsilon,cont}^{crit} = \mu_S M_S^{crit}$ such that above this critical threshold no positive equilibrium can exists, only the trivial equilibrium, i.e. $\mathbf{0}_{\mathbb{R}^7}$. We are not able to derive a formula for M_S^{crit}, and thus for $\Lambda_{\varepsilon,cont}^{crit}$, but we can solve (S13), page 10, to find it.

Assume $\varepsilon \in [0, \varepsilon_{\max}]$, and Λ, the release rate, greater than $\Lambda_{cont,\varepsilon}^{crit}$, such that according to the previous reasoning, only the trivial equilibrium exists, $\mathbf{0}_{\mathbb{R}^7}$, that is also LAS. Thanks to the Theory of Monotone Cooperative system [2], and following [3, Theorem 6] or [4, Theorem 1], it is straightforward to deduce that $\mathbf{0}_{\mathbb{R}^7}$ is not only LAS but it is GAS when $\varepsilon \leq \varepsilon_{\max}$. The equilibrium $\mathbf{0}_{\mathbb{R}^7}$ being GAS for the auxiliary Monotone system (S11), it is also GAS for system (S10), for $\Lambda > \Lambda_{cont,\varepsilon}^{crit}$.

References

1. Berman A, Plemmon RJ. Nonnegative Matrices in the Mathematical Sciences. Academic Press, New York, San Francisco, London.; 1979

2. Smith H. Monotone dynamical systems: An introduction to the theory of competitive and cooperative systems. American Mathematical Society, Providence, RI.; 2008.

3. Anguelov R, Dumont Y, Lubuma J. Mathematical modeling of sterile insect technology for control of anopheles mosquito. Comput. Math. Appl. 2012;64:374–389.

4. Anguelov R, Dumont Y, Yatat Djeumen IV. Sustainable vector/pest control using the permanent sterile insect technique. Mathematical Methods in the Applied Sciences 2020; 43(18):10391–10412.
